# Enhancing Thermal Protection in Lithium Batteries with Power Bank-Inspired Multi-Network Aerogel and Thermally Induced Flexible Composite Phase Change Material

**DOI:** 10.1007/s40820-024-01593-0

**Published:** 2025-02-26

**Authors:** Zaichao Li, Feng Cao, Yuang Zhang, Shufen Zhang, Bingtao Tang

**Affiliations:** State Key Laboratory of Fine Chemicals, Frontiers Science Center for Smart Materials Oriented Chemical Engineering, Dalian University of Technology, Dalian, 116024 People’s Republic of China

**Keywords:** Lithium-ion battery thermal runaway, Thermal protection material, Multinetwork aerogel, Flexible composite phase change material

## Abstract

Thermal runaway (TR) is considered a significant safety hazard for lithium batteries, and thermal protection materials are crucial in mitigating this risk. However, current thermal protection materials generally suffer from poor mechanical properties, flammability, leakage, and rigid crystallization, and they struggle to continuously block excess heat transfer and propagation once thermal saturation occurs. This study proposes a novel type of thermal protection material: an aerogel coupled composite phase change material (CPCM). The composite material consists of gelatin/sodium alginate (Ge/SA) composite biomass aerogel as an insulating component and a thermally induced flexible CPCM made from thermoplastic polyester elastomer as a heat-absorbing component. Inspired by power bank, we coupled the aerogel with CPCM through the binder, so that CPCM can continue to ‘charge and store energy’ for the aerogel, effectively absorbing heat, delaying the heat saturation phenomenon, and maximizing the duration of thermal insulation. The results demonstrate that the Ge/SA aerogel exhibits excellent thermal insulation (with a temperature difference of approximately 120 °C across a 1 cm thickness) and flame retardancy (achieving a V-0 flame retardant rating). The CPCM exhibits high heat storage density (811.9 J g^−1^), good thermally induced flexibility (bendable above 40 °C), and thermal stability. Furthermore, the Ge/SA-CPCM coupled composite material shows even more outstanding thermal insulation performance, with the top surface temperature remaining at 89 °C after 100 min of exposure to a high temperature of 230 °C. This study provides a new direction for the development of TR protection materials for lithium batteries.

**Supplementary Information:**

The online version contains supplementary material available at 10.1007/s40820-024-01593-0.

## Highlights

- The prepared Ge/SA biomass aerogel with multiple crosslinked networks have excellent flame retardancy and thermal insulation properties.
- The prepared SAT/TPEE/EG composite phase change material (CPCM) has a thermal storage density as high as 811.9 J g^–1^ and good flame retardancy.
- In the composite material of CPCM coupled with aerogel, the CPCM continuously absorbs heat for the aerogel, thus maximizing heat transfer and spreading.

**Supplementary Information:**

The online version contains supplementary material available at 10.1007/s40820-024-01593-0.

## Graphical abstract

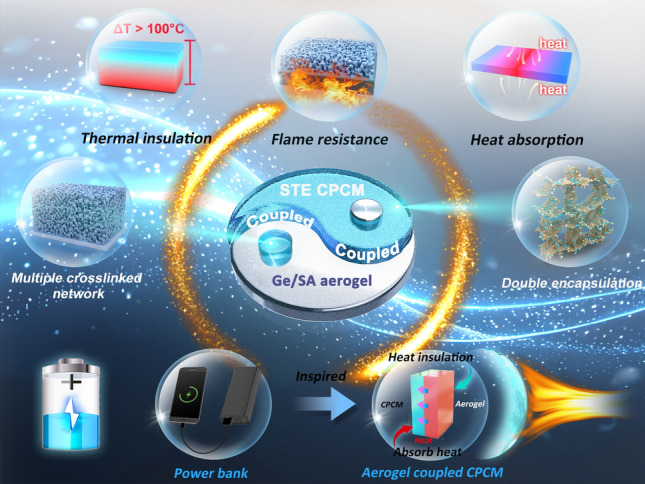

## Introduction

Lithium-ion (Li-ion) batteries, due to their high energy density, long cycle life, and lack of memory effect, are widely used in 3C electronics, aerospace, and new energy fields [1–5]. However, in recent years, incidents of fires and explosions caused by thermal runaway (TR) of Li-ion batteries have occurred frequently [6]. This is because, when a single Li-ion battery undergoes overcharging, short-circuiting, or other abusive conditions, a large amount of heat can be generated in a short period. When the battery temperature reaches 100–130 °C, the separator begins to melt, leading to an internal short circuit, which in turn triggers TR, posing a high risk of fire or even explosion. What is more concerning is that to meet high energy demands, multiple individual cells are often assembled into a battery module [7]. Once TR occurs in a single cell within the module, the heat may spread to adjacent cells through thermal conduction, potentially causing TR of the entire battery module. Compared to a single cell, the TR of an entire battery module releases more heat and poses a greater hazard, possibly leading to catastrophic fire or explosion accidents [8]. Therefore, it is crucial to develop suitable thermal protection materials to enhance the thermal safety of the entire battery module [9]. Currently, thermal protection materials applied to Li-ion battery TR are mainly divided into two categories [10]: one type is thermal insulation materials, such as aerogels [11, 12]; the other type is heat-absorbing materials, such as phase change materials (PCMs) [13, 14]. These two types of materials delay the accumulation and transfer of heat through insulation or heat absorption, thereby providing more time for cooling and safely handling Li-ion batteries.

In recent years, aerogel materials, as a representative of thermal insulation materials, have been considered the most suitable for thermal insulation due to their low thermal conductivity, ultra-lightweight, and low bulk density [15]. For example, inorganic aerogels (such as silica aerogel) have extremely low thermal conductivity, but their difficult molding process limits their wide application [16]. On the other hand, organic aerogels (such as polyurethane and polyimide) are easier to mold, but their synthesis process is complex and involves toxic solvents and raw materials, which can lead to environmental pollution [17–19]. In contrast, aerogels based on natural biomass have garnered widespread attention from researchers due to their abundant availability, low cost, and environmental friendliness [20]. However, biomass aerogels also have drawbacks. For instance, sodium alginate (SA) aerogels, while having advantages such as being non-flammable, good smoke suppression, and non-toxic, suffer from poor mechanical properties [21]. Similarly, gelatin (Ge) aerogels derived from collagen exhibit excellent mechanical properties but are flammable and have poor smoke suppression [22]. Therefore, developing high-performance biomass-based composite aerogels that possess both good mechanical properties and flame retardancy is of great significance for their application in Li-ion battery thermal protection, but this remains a significant challenge.

Among heat-absorbing materials, PCMs are particularly suitable for thermal protection since they can absorb a large amount of heat during phase transitions and have a high latent heat density [23]. PCMs refer to substances that achieve temperature regulation within a specific range by absorbing heat (or cold) from the environment during phase transitions or by releasing heat (or cold) into the environment during these transitions [24, 25]. However, most PCMs currently used for Li-ion battery thermal management are organic solid–liquid PCMs. Although they exhibit stable chemical properties, their flammability is a critical drawback [26]. During Li-ion battery TR, the flammable nature of organic PCMs may increase the thermal hazards of the entire battery system, thereby raising the risk of fire and explosion. In contrast, inorganic solid–liquid PCMs are not only entirely non-flammable but also more cost-effective and possess higher latent heat values [27]. In recent years, an increasing number of researchers have applied inorganic solid–liquid PCMs in thermal management systems for Li-ion batteries [28–31]. Among various inorganic solid–liquid PCMs, sodium acetate trihydrate (SAT) has attracted widespread attention due to its suitable phase transition temperature and relatively high phase change enthalpy. In SAT, heat is stored primarily at two temperatures: latent heat at 58 °C and thermochemical heat at 106–140 °C. These two temperature stages not only provide early warning of TR but also help suppress heat propagation when TR occurs. However, SAT also has some problems, such as the leakage problem during the phase transition, the crystalline rigidity problem (the crystalline rigidity refers to the fact that the crystalline structure formed during the phase transition will lead to a sharp increase in the rigidity of the phase transition material itself), and the molding problem after encapsulation, which limit the practical application of SAT. At present, some researchers have tried to use porous materials or polymers to encapsulate SATs to solve the problems of leakage or rigidity, and these excellent works have undoubtedly solved some of the problems of SATs to some extent [32–35]. However, the leakage problem, the crystalline rigidity problem, and the molding problem after encapsulation of SAT have not been solved at once. Therefore, it is important for the application of SAT in the field of thermal protection to solve all the problems of SAT at once.

In addition to the inherent issues of the two major categories of thermal protection materials, there are also challenges in their application to thermal protection. For example, as the energy density of Li-ion batteries continues to increase, aerogels may experience thermal saturation, at which point they can no longer block all the excess heat. Phase change heat-absorbing materials face similar issues-once their latent heat and thermochemical heat storage capacity are exhausted, these materials can no longer maintain a constant temperature, thus failing to prevent the transmission and spread of excess heat. Therefore, finding ways to delay the onset of thermal saturation and to maximize the delay of heat diffusion is of great significance for their application in thermal protection, as it provides more time for the safe handling of batteries.

**Scheme 1:**
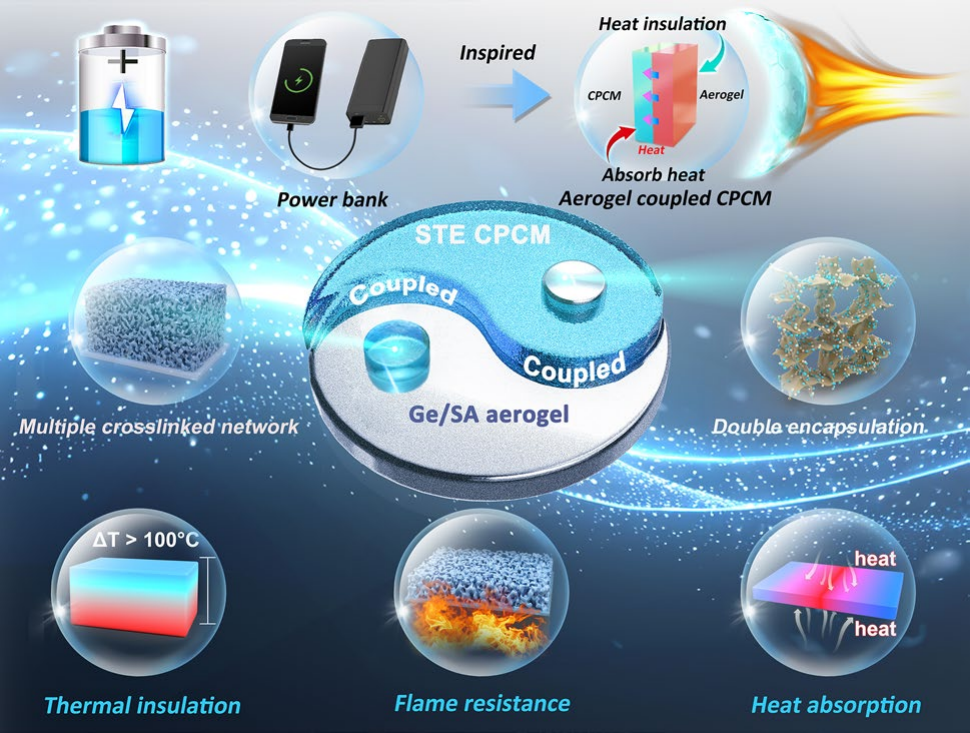
Train of thought of this work

With the aim of addressing these issues, we present a biomass-based composite aerogel with a multiscale crosslinked network, using Ge and SA as the aerogel matrix, with glutaraldehyde (GLU) and boric acid (BA) as dual crosslinking agents. Simultaneously, SAT was used as the PCM, with thermoplastic polyester elastomer (TPEE) and expanded graphite (EG) as encapsulating materials, successfully creating a thermally induced CPCM. Inspired by power banks charging electronic devices, we proposed coupling the Ge/SA composite aerogel with SAT/TPEE/EG (STE) through a binder. This configuration allows STE to continuously "charge and store energy" for the Ge/SA aerogel by absorbing and dissipating heat, thereby delaying the occurrence of thermal saturation phenomenon, maximally improving the thermal insulation time of the composite material, delaying the heat diffusion, and making it well used in the field of Li-ion battery thermal protection (Scheme 1). The chemical crosslinked network from Ge and GLU provides mechanical strength, while the hydrogen-bonded network from SA and BA enhances flame retardancy. This creates a biomass composite aerogel with both excellent mechanical properties and flame retardancy. Additionally, through the elastic behavioral support properties of TPEE and the porous adsorption properties of EG, CPCMs with good shape stability, thermotropic flexibility and the ability to be shaped after being encapsulated are constructed through the strategy of dual encapsulation. Finally, the materials were coupled with a binder, maximizing thermal protection. The results show that the Ge/SA-STE coupled material maintains a top surface temperature of only 89 °C even after being subjected to 230 °C for 100 min. In addition, the coupling material also has good flame retardant properties, which is very suitable for application in the field of thermal protection of Li-ion batteries, to slow down the transmission and spread of heat when the Li-ion battery TR.

## Experimental Section

### Preparation Method

Preparation of Ge/SA aerogel: First, 1.0 g of Ge and 0.4 g of SA were added to 20 mL of acetic acid solution (0.2 mol L^−1^). The mixture was stirred using a magnetic stirrer at 70 °C and 500 r min^−1^. After 1 h of stirring, both Ge and SA were completely dissolved in the acetic acid solution. Then, 0.01 g of BA was added for crosslinking, followed by an additional 2 h of stirring. Afterward, 167 μL of GLU solution was added for further crosslinking, and the solution was stirred for an additional 0.5 h. The resulting solution was then poured into a polytetrafluoroethylene (PTFE) mold and pre-frozen in a -30 °C constant temperature and humidity chamber for 12 h. Finally, the sample was freeze-dried for 24 h in a freeze dryer (FDU-1200, Tokyo Rikakikai, Japan) to remove the solvent, yielding the Ge/SA multinetwork aerogel.

Preparation of STE CPCM: First, 4 g of TPEE was dissolved in 100 mL of dichloromethane solution at 50 °C, with magnetic stirring at 500 r min^−1^ at room temperature for 30 min to ensure complete dissolution of TPEE. Next, 22.0 g of SAT was heated in a water bath at 60 °C to its liquid state, followed by the addition of 0.7 g of EG for adsorption. After 1 h of adsorption, the adsorbed powder was collected, ground into fine powder using a ball mill, and then mixed with the previously dissolved TPEE solution. The mixture was stirred with a magnetic stirrer at 500 r min^−1^ for 1 h to ensure uniform mixing. The solution was then placed in a vacuum drying oven at 50 °C for 2 h for adsorption. Afterward, the mixture was left in the air for 24 h to allow the dichloromethane solvent to evaporate. Finally, the sample was placed in a vacuum drying oven for 24 h to remove any remaining dichloromethane, resulting in the STE CPCM. The quality of the initial addition of each component of the CPCM is detailed in Table S1.

Preparation of Ge/SA-STE coupled material: A certain amount of adhesive was applied to the surface of the STE material, and the aerogel was then pressed onto it. The coupled material was cured at room temperature for 48 h to obtain the final product.

### Thermal Insulation Performance Test

A high-efficiency constant temperature heating plate (35 × 21 × 5 mm^3^, 24 V, Kaitai Instrument Co., Ltd., China) was used to simulate a high-temperature environment to evaluate the thermal insulation performance of the composite material. First, the material to be evaluated was placed on a ceramic heating plate, and the two were fixed using a mold. A K-type thermocouple (Toprei Electronics Co., Ltd., accuracy: 0.6 °C, China) was placed on both sides of the test sample, and a DC regulated power supply (IT6722A, Itech Electronics Co., Ltd., China) was used to power the ceramic heating plate. The maximum current of the power supply was set to 3 A, and the maximum voltage was set to 30 V to ensure full-power heating of the plate. In approximately 30 s, the temperature of the heating plate increased from room temperature (25 °C) to around 230 °C. The DC power supply operated in constant current-constant voltage (CC-CV) mode, a two-stage power control algorithm that ensures stable power supply to the heating plate efficiently and safely. In the constant current stage, power is supplied rapidly by limiting the current, while in the constant voltage stage, fixed voltage is maintained to prevent overvoltage, balancing power supply efficiency with equipment safety. During the entire test, a temperature data logger (MIK-R5000C, Hangzhou Meacon Automation Technology Co., Ltd., China) was used to record the temperature difference on both sides of the sample to assess its thermal insulation performance.

For infrared thermal imaging testing of the sample’s insulation performance (as shown in Fig. S1), the sample was placed on a heating plate at 200 °C, and infrared thermal images were captured at different time intervals to visually display the temperature distribution and insulation performance of the sample.

### Li-Ion Battery Thermal Runaway Test

The thermal insulation performance of the thermal protective material was further validated through TR experiments of Li-ion batteries. A prismatic Li-ion battery with a single cell capacity of 30 Ah, a nominal voltage of 3.2 V, and dimensions of 100 × 140 × 20.5 mm^3^ was used for the TR tests, with specific battery parameters shown in Table S2. TR was triggered by overcharging the Li-ion battery at a 0.5 C rate, and it was considered to occur when three consecutive temperature rise rates of ≥ 3 °C s^−1^, fire, or explosion were observed. The Li-ion battery pack was secured with stainless steel plates and four screws. To monitor temperature changes, a K-type thermocouple (Topray Electronics Co., Ltd., China) was attached to the center of the Li-ion battery surface, and another K-type thermocouple was attached to the backside of the thermal protective material (the side away from the Li-ion battery) to record temperature changes. The thermal protective material, 1 cm thick, was placed in direct contact with the Li-ion battery surface. Before testing, the Li-ion battery was charged to 3.9 V at a 0.5 C rate, followed by constant voltage charging until the current dropped to 1 A to ensure a state of charge (SOC) of 100% for all cells. The C-rate is a standardized parameter used to quantify the battery's charge and discharge rates, representing the current relative to its nominal capacity. Temperature variations during TR were recorded using a data logger, and the entire process was captured with a camera. Data logging and video recording commenced as the Li-ion battery started overcharging. For comparison, a blank group consisting of a Li-ion battery without thermal protective material was also tested. A K-type thermocouple (Topray Electronics Co., Ltd., China) was attached to the center of the bare battery surface to record temperature changes, with all other test conditions identical to those used for the battery with thermal protective material.

### Uncertainty Analysis

The primary sources of uncertainty in temperature measurement come from the precision of the thermocouple and the temperature logger. During the experiment, the temperature range was between 20 and 230 °C, with a thermocouple measurement error of 0.3% (${\sigma }_{T}$) and a temperature logger measurement error of 0.2% (${\sigma }_{D}$). The total error ($\sigma$) was calculated using Eq. (1) [36]:

1 $$\begin{array}{c}\sigma =\pm \sqrt{{{\sigma }_{T}}^{2}+{{\sigma }_{D}}^{2}}\#\end{array}$$

The total error (σ) was calculated to be 0.36%, meaning that within the temperature range of 20–230 °C, the maximum total temperature error was 0.756 °C.

## Results and Discussion

### Construction of the Ge/SA Multinetwork Composite Aerogel System

**Fig. 1:**
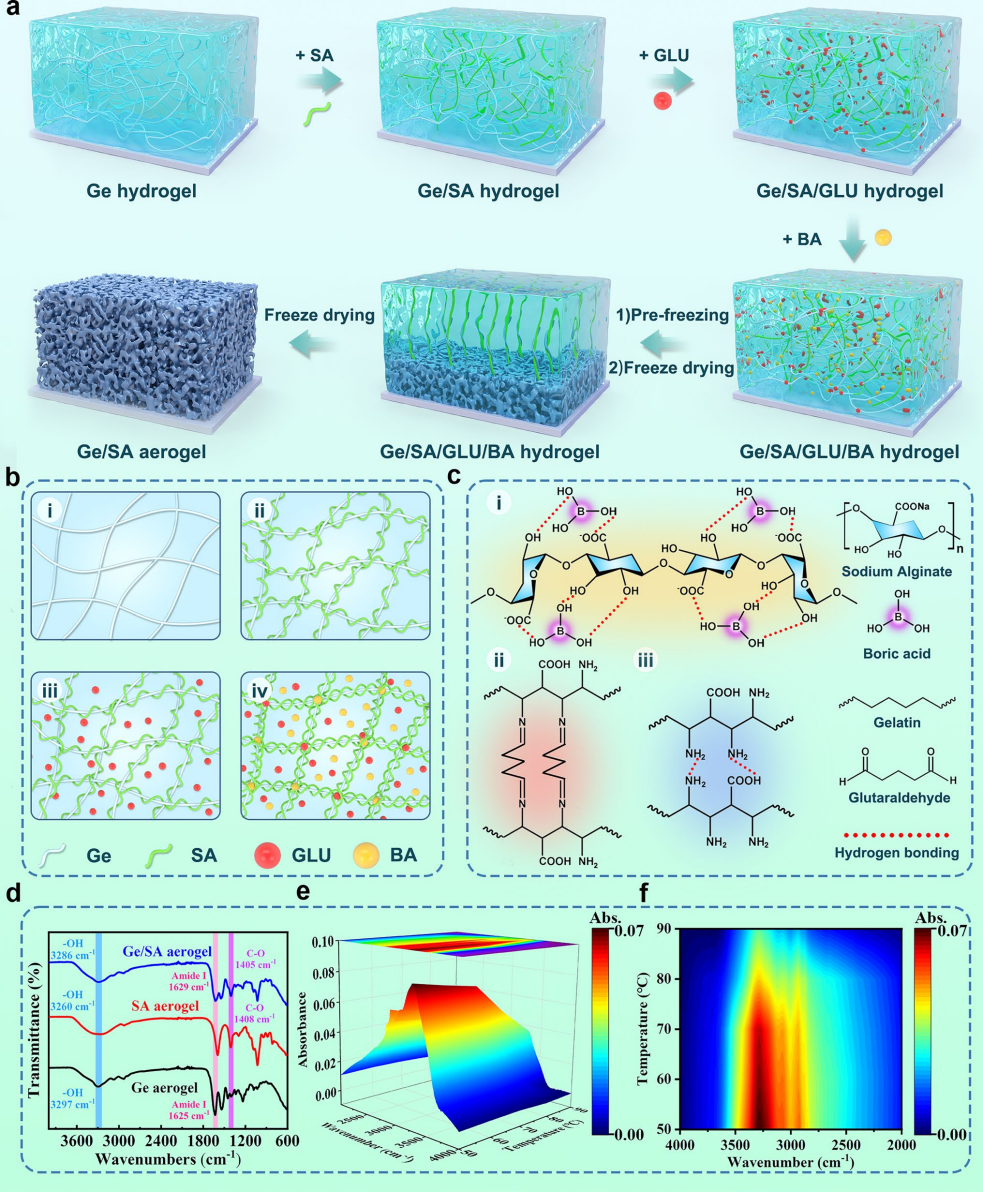
**a** Schematic illustration of the preparation of Ge/SA composite aerogel. **b** Multinetwork system formed during the construction of Ge/SA aerogel. **c** Possible mechanism of the bonding within the multinetwork crosslinked structure of Ge/SA aerogel. **d** FT-IR spectra of Ge aerogel, SA aerogel, and Ge/SA aerogel. **e** and **f** In-situ variable temperature infrared spectra and corresponding contour plots of Ge/SA aerogel

As shown in Fig. 1a, b, we prepared a composite aerogel with a multinetwork crosslinked structure using the freeze-drying method. Specifically, Ge was first fully dissolved in an acetic acid solvent, followed by the addition of a certain amount of SA. Once completely dissolved, two crosslinking agents, GLU and BA, were sequentially added for crosslinking. After the crosslinking process was completed, the mixture was pre-frozen for 24 h and then freeze-dried for a certain period to obtain the Ge/SA multinetwork aerogel. The multinetwork structure mainly consists of three parts (Fig. 1c): the first network is a hydrogen-bonded crosslinked network formed between Ge molecules; the second is a chemical crosslinked network formed by the Schiff base reaction between Ge and GLU; the third crosslinked network is a hydrogen-bonded network formed between the –OH and –COOH groups of SA and the –OH groups in BA. The crosslinked network formed by Ge mainly provides the mechanical strength of the aerogel, while the network formed by SA primarily enhances the flame retardancy of the aerogel.

Fourier transform infrared spectroscopy (FT-IR) analysis was conducted to verify the formation of multinetwork structures within Ge/SA aerogel, as shown in Fig. 1d, f. Figure 1d presents the FT-IR spectra of Ge before and after modification. For the unmodified Ge, the band at 3296 cm^−1^ corresponds to the Amide A band, related to the vibrational absorption of –NH and –OH groups within Ge. The band at 1629 cm^−1^ is associated with the Amide I band, a characteristic band for the stretching vibrations of C = O and Schiff base (C = N). The Amide II band, near 1540 cm^−1^, is attributed to the stretching vibration of C–N and the bending vibration of N–H in proteins. The Amide III band, located around 1235 cm^−1^, is linked to the stretching vibration of C–N and bending vibration of N–H in Ge. Following the addition of GLU as a crosslinker and SA, the changes in functional groups were primarily reflected in the absorption peaks of amino and aldehyde groups. As shown in Fig. 1d, in the spectra of crosslinked Ge/SA, the absorption peaks corresponding to Amide I, Amide II, and Amide III bands shifted to 1628, 1537, and 1238 cm^−1^, respectively, with a reduction in intensity. This suggests that a crosslinking reaction occurred between the aldehyde groups of GLU and the amino groups in Ge, leading to the formation of a Schiff base [37]. Additionally, the shifts in the absorption peaks of Amide A, Amide II, and Amide III bands confirm the presence of hydrogen bonding within the Ge molecules. For SA, the broad characteristic peak at 3260 cm^−1^ corresponds to the -OH group, while the characteristic absorption peaks at 1594 and 1408 cm^−1^ are associated with the –COO and C–O stretching vibrations of SA, respectively, and the peak at 1029 cm^−1^ is attributed to the C–O–C group. After the incorporation of BA and Ge, significant changes were observed in the absorption peaks of –OH and –COO. As shown in Fig. 1d, in the spectra of crosslinked Ge/SA, the –OH absorption peak shifted to 3286 cm^−1^, while the −COO and C−O absorption peaks shifted to 1627 and 1405 cm^−1^, respectively, indicating that the crosslinking between BA and SA is mediated by hydrogen bonding. To further confirm the existence of hydrogen bonding in the aerogel, variable temperature FT-IR characterization of the Ge/SA aerogel was performed (Figs. 1e, f and S2). As the temperature increased from 50 to 90 °C, the intensity of the absorption peak at 3286 cm^−1^ (-OH absorption peak) gradually decreased, indicating the progressive dissociation of hydrogen bonds with rising temperature. These results strongly corroborate the presence of hydrogen bonding interactions within the Ge/SA multinetwork aerogel.

**Fig. 2:**
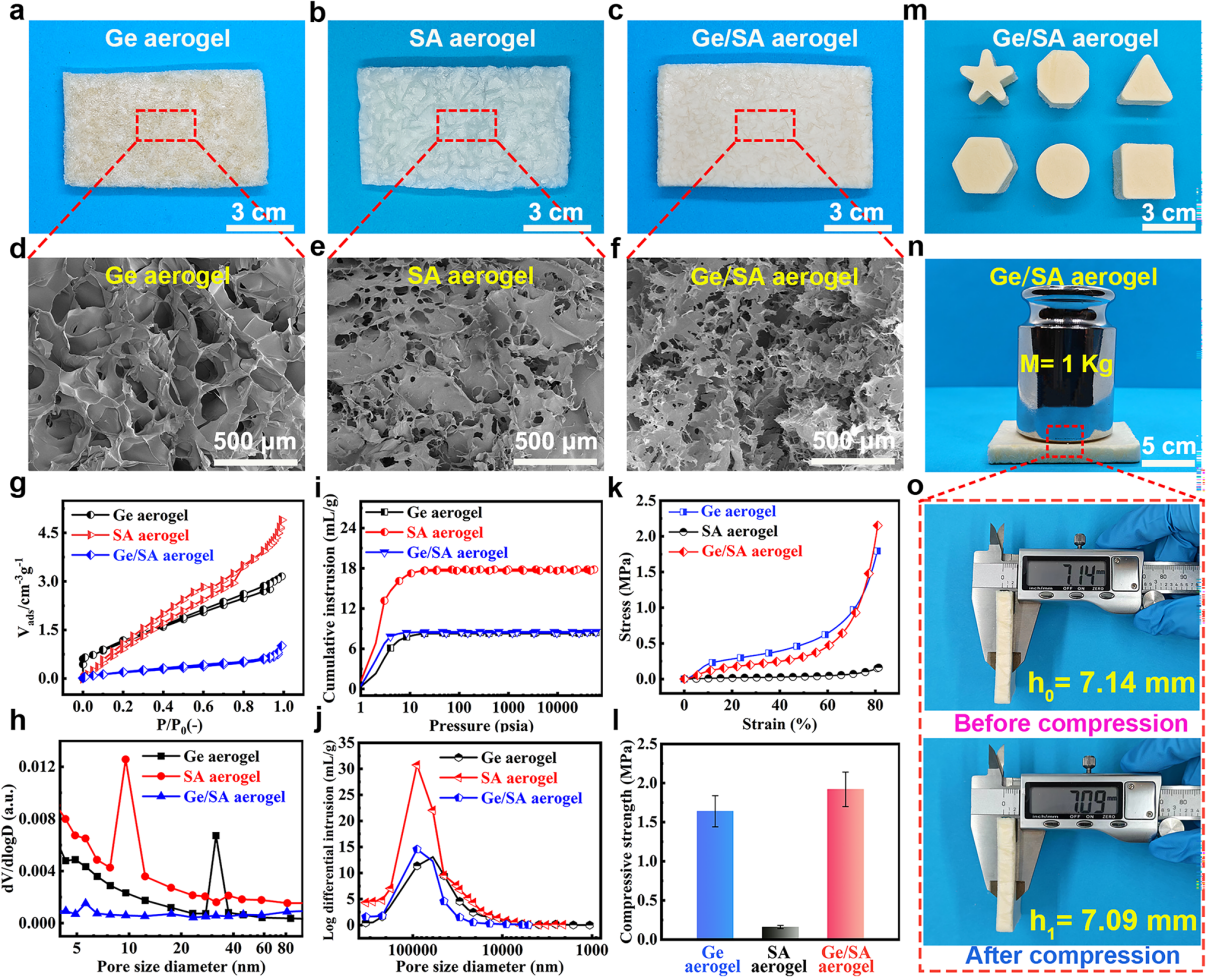
**a–c** Photographs and **d–f** SEM images of Ge, SA, and Ge/SA composite aerogels.** g** and** h** Nitrogen adsorption–desorption isotherms and corresponding pore size distribution curves of Ge, SA, and Ge/SA composite aerogels. ** i** and **j** Cumulative mercury intrusion curves and corresponding pore size distribution curves of Ge, SA, and Ge/SA composite aerogels. **k** and** l** Compressive stress–strain curves and corresponding compressive strength comparison charts of Ge, SA, and Ge/SA composite aerogels. **m** Photographs of Ge/SA composite aerogels in different shapes. **n** and **o** Visual representation of the mechanical properties of Ge/SA aerogel

The physical images of the three types of aerogels prepared via freeze-drying are shown in Fig. 2a–c. To characterize the morphological details of the aerogels, SEM characterization was conducted on all three aerogels (Fig. 2d, f). There is a well-developed porous structure in all three aerogels shown in the figures. In order to explore the pore structure of the three aerogels, nitrogen adsorption–desorption analysis and mercury intrusion porosimeter were employed to conduct a comprehensive pore size analysis. The results are depicted in Fig. 2g–j (specific test device is shown in Fig. S3a, b), revealing that the pore size distribution of the three aerogels primarily falls within the range of 20–200 μm, suggesting that the composite aerogels are mainly composed of macropores.

Composite aerogels were tested for their mechanical properties by compression stress–strain tests, as shown in Fig. 2k, l (specific test device is shown in Fig. S3c). There is a relatively stable trend for linear elastic behavior in all three aerogels under low strain. The compressive strength of the Ge/SA aerogel surpasses that of the Ge and SA composite aerogels (Fig. 2l). Ge/SA aerogel material exhibits significant mechanical enhancement due to the formation of multiple crosslinked networks. To visually observe the mechanical performance of the Ge/SA aerogel, a 1 kg weight was placed on it, and the thickness change before and after compression was measured, with the results presented in Fig. 2n, o. The thickness remained nearly unchanged, indicating excellent compressive resistance. Furthermore, to verify that the crosslinked network formed by Ge is primarily responsible for providing the mechanical strength of the Ge/SA composite aerogel, the compression stress–strain behavior of the Ge aerogel without GLU was also tested, as shown in Figs. S4 and S5. The compressive strength of the Ge aerogel with GLU is nearly double that of the one without, indicating that the crosslinked network formed between Ge and GLU is the primary contributor to the mechanical strength of the Ge/SA composite aerogel. The shape of the Ge/SA composite aerogel depends on the mold used during the freezing process, as illustrated in Fig. 2m. Thanks to the shape designability during the freezing and freeze-drying processes, various aerogel shapes can be successfully obtained using different molds. As shown in Fig. S6, the Ge/SA composite aerogel can be stably fixed onto a leaf without deformation, attributed to its ultralight weight.

**Fig. 3:**
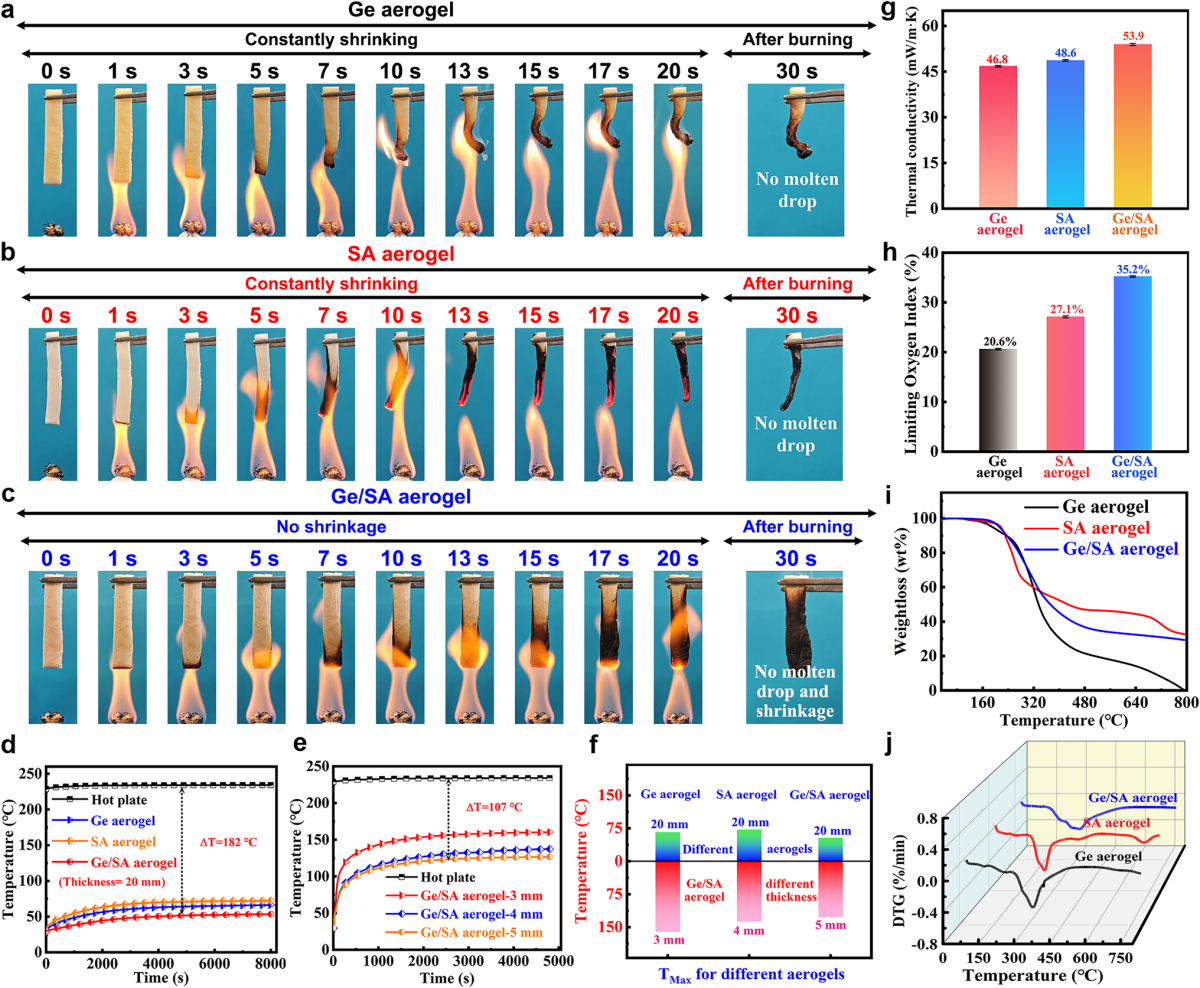
**a-c** Time-lapse photographs of the combustion process of Ge, SA, and Ge/SA composite aerogels. **d** and **e** Thermal insulation performance testing of different aerogels on a heating plate and their corresponding temperature curves over time. **f** Comparison of the thermal insulation performance of different aerogels. **g** Thermal conductivity and **h** limiting oxygen index of Ge, SA, and Ge/SA composite aerogels. **i** and **j** TG and DTG curves of Ge, SA, and Ge/SA composite aerogels

In evaluating the application of thermal protective materials, flame retardancy and fire resistance are crucial factors. Materials that retard flame spread can provide valuable time for rescue operations when there is a fire. Therefore, we tested the flame retardancy and fire resistance of the aerogels (Fig. 3a–c). As seen from the figures, both Ge and SA aerogels exhibit continuous shrinkage and can be ignited as the burning time increases. In contrast, the Ge/SA composite aerogel shows almost no shrinkage and cannot be ignited under the same burning conditions, only exhibiting surface charring. This indicates that the Ge/SA composite aerogel possesses excellent flame retardancy and fire resistance. To further assess their flame retardancy, we conducted LOI tests, with the results shown in Fig. 3h. The LOI of the Ge aerogel is only 20.6%, categorizing it as a flammable material. In comparison, the LOI of the SA aerogel and the Ge/SA composite aerogel are 27.1% and 35.2%, respectively, classifying them as flame-retardant materials. This improved flame retardancy is primarily attributed to the crosslinked network formed between SA and BA, which provides the Ge/SA composite aerogel with good flame-retardant properties. To verify this conclusion, we also tested the LOI of non-crosslinked SA aerogel, as shown in Fig. S7. The results show that the LOI of non-crosslinked SA aerogel is only 21.5%, whereas the LOI increases to 27.1% after BA crosslinking. This suggests that BA not only acts as a crosslinking agent but also as a flame retardant, with the crosslinked network formed between it and SA providing the Ge/SA composite aerogel with excellent flame-retardant properties. Additionally, we performed a vertical burning test on the Ge/SA composite aerogel, which achieved the highest flame-retardant rating of V-0 (Table S3).

Thermal insulation performance is another important indicator for evaluating the application of thermal protective materials. Therefore, we placed the three types of aerogels on a heating plate at around 230 °C to assess their thermal insulation performance, with the results shown in Fig. 3d-f. It can be seen that when the aerogel thickness is 2 cm, the Ge/SA composite aerogel exhibits the best thermal insulation effect among the three, with a temperature difference of 182 °C between the top and bottom surfaces. Even when the thickness is reduced to 5 mm, the temperature difference remains at 107 °C, indicating excellent thermal insulation performance. This outstanding thermal insulation is primarily due to its ultra-low thermal conductivity; as shown in Fig. 3g, the thermal conductivity of the Ge/SA composite aerogel is only 53.9 mW m^−1^ K^−1^. Additionally, the presence of multiple crosslinked networks greatly hinders the rapid transfer of heat, thus providing good thermal insulation performance. Moreover, aerogels' thermal stability is another critical factor. We measured the thermal stability of the three types of aerogels (Fig. 3i, j). It can be observed that there is no weight loss from room temperature to around 150 °C, indicating excellent thermal stability. To visually demonstrate the superiority of the Ge/SA composite aerogel compared to the other two aerogels, we compared the flame retardancy, thermal insulation, and compressive properties of the aerogels (Fig. S8). The Ge/SA composite aerogel outperforms the single-network aerogels in terms of flame retardancy, thermal insulation, and compressive properties, highlighting the advantages of the composite aerogel constructed from multiple crosslinked networks.

### Construction of the SAT/TPEE/EG Elastomer-Based CPCM System

**Fig. 4:**
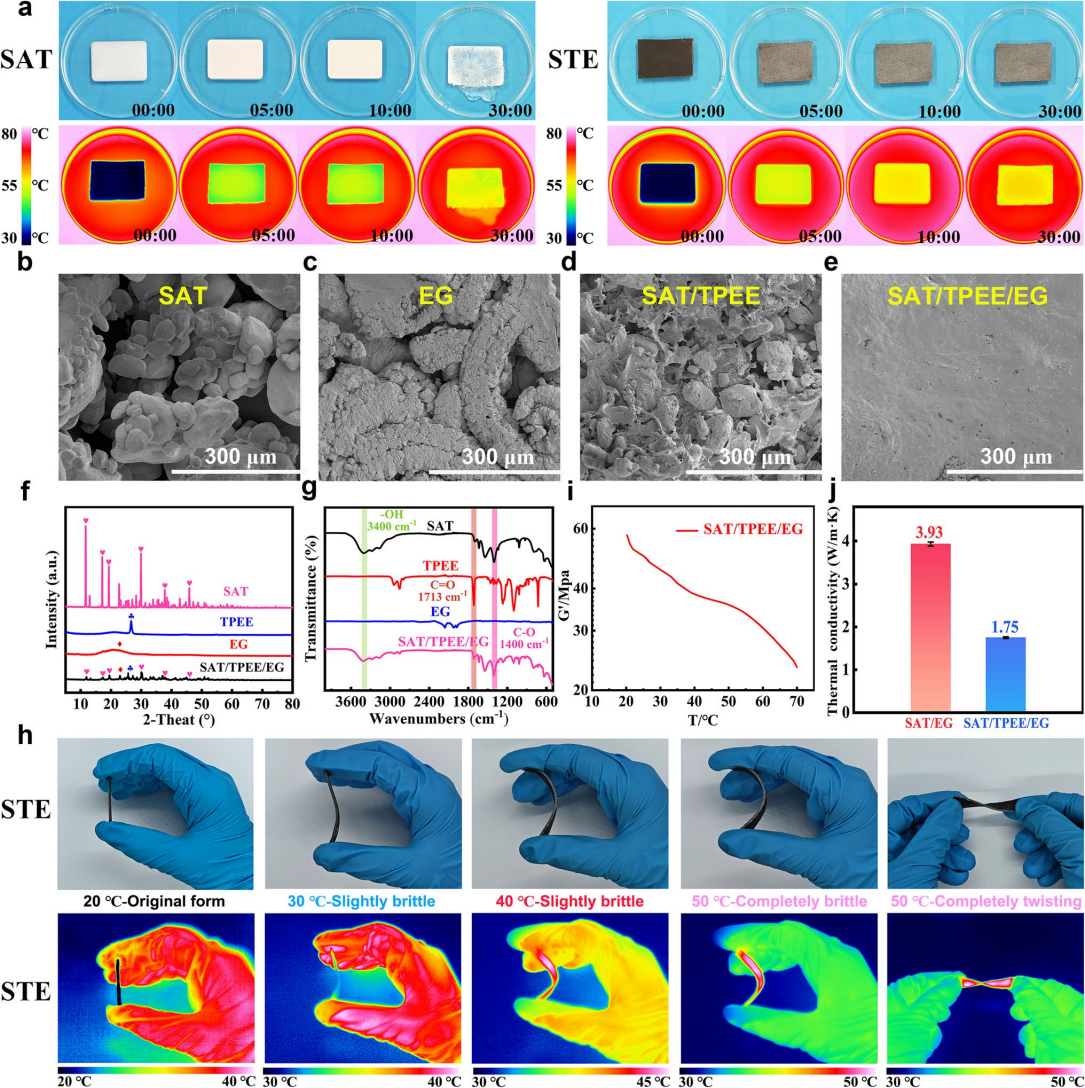
**a** Digital photographs of SAT and SAT/TPEE/EG, along with their corresponding infrared thermal imaging photos. **b−e** SEM images of SAT, EG, SAT/TPEE, and SAT/TPEE/EG. **f−g** XRD spectra and FT-IR spectra of SAT, TPEE, EG, and SAT/TPEE/EG. **h** Digital photographs and corresponding infrared thermal imaging photos of SAT/TPEE/EG at different temperatures. **i** Storage modulus of SAT/TPEE/EG at different temperatures. **j** Comparison of thermal contact resistance between SAT/EG and SAT/TPEE/EG

As mentioned in the introduction, although aerogels possess excellent thermal insulation properties, they will eventually experience thermal saturation. Therefore, to extend the insulation duration of aerogels, other methods are needed to "store energy" and help slow down the onset of thermal saturation. PCMs can absorb a large amount of heat within a specific temperature range while maintaining their temperature almost unchanged. Therefore, we developed the SAT/TPEE/EG elastomer-based CPCM to serve as an "energy storage" material for the Ge/SA composite aerogel, helping to mitigate thermal saturation. We prepared the SAT/TPEE/EG CPCM using a "dual encapsulation" strategy, as shown in Fig. S9. First, the porous adsorption effect of EG was used to adsorb liquid SAT into its pores. Then, the SAT-adsorbed EG powder was crushed and added to a fully dissolved TPEE solution. The SAT/EG powder was then vacuum-adsorbed into the crosslinked network of the elastomer TPEE, thus forming the SAT/TPEE/EG CPCM. The CPCM prepared using this "dual encapsulation" strategy exhibits excellent shape stability. As shown in Fig. 4a, after heating for 30 min, the SAT had already leaked, while the SAT/TPEE/EG maintained its shape stability without any leakage. To further verify its excellent shape stability, we tested the leakage rates of SAT/TPEE and SAT/TPEE/EG (Fig. S10 is a schematic diagram of the leak test), with the results shown in Table S4. It can be seen that the single-encapsulated SAT/TPEE had a leakage rate of 27.821% after 120 min, while SAT/TPEE/EG exhibited no leakage at all, indicating the significant superiority of the dual encapsulation strategy over single encapsulation.

Figures 4b-e and S11 present the scanning electron microscopy (SEM) images of various materials, where it is evident that the SAT/TPEE/EG composite exhibits a smooth surface, indicating effective encapsulation of SAT within the matrix. To further explore the structural features of the CPCM, X-ray diffraction (XRD) and FT-IR analyses were conducted (Fig. 4f, g). The characteristic peaks of SAT, TPEE, and EG are all present in the SAT/TPEE/EG composite, suggesting that these components are combined through physical interactions, with no evidence of chemical reactions.

The most important reason why we chose TPEE as the support skeleton is to achieve the flexibility of CPCM, TPEE is widely regarded as a good flexible material with excellent mechanical properties and flexibility at low temperatures because the internal rotational resistance of the ether bond (C–O–C) in the soft segments is much lower than that of the C–C bond, thus it has good flexibility [36]. Benefiting from this advantage, we incorporated SAT/EG powder to take advantage of the fact that TPEE combines good flexibility and encapsulation properties, thus preparing SAT/TPEE/EG with thermotropic flexibility. Photographs illustrating the flexible behavior of SAT/TPEE/EG at various temperatures are shown in Fig. 4h. It is clear that the composite does not bend at 20 °C, exhibits slight bending at 30 °C, and demonstrates significant flexibility within the 40–50 °C range. This flexibility enhances the interface fit with the aerogel, thereby improving thermal protection efficiency. To quantify the flexibility of SAT/TPEE/EG, a rheometer was used to measure the modulus variation of material, with the results depicted in Fig. 4i. As the temperature increases, the storage modulus decreases progressively, indicating that the material exhibits good flexibility at specific temperatures. This flexibility is crucial in reducing thermal contact resistance. We measure the thermal contact resistance of SAT/EG and SAT/TPEE/EG, with the results shown in Fig. 4j. The thermal contact resistance values for the rigid SAT/EG and flexible SAT/TPEE/EG were 3.93 and 1.75 °C W^−1^. Significantly lower thermal contact resistance is evident with SAT/TPEE/EG. Consequently, the thermally induced flexibility of SAT/TPEE/EG reduce the roughness of the surface, thereby lowering thermal contact resistance.

**Fig. 5:**
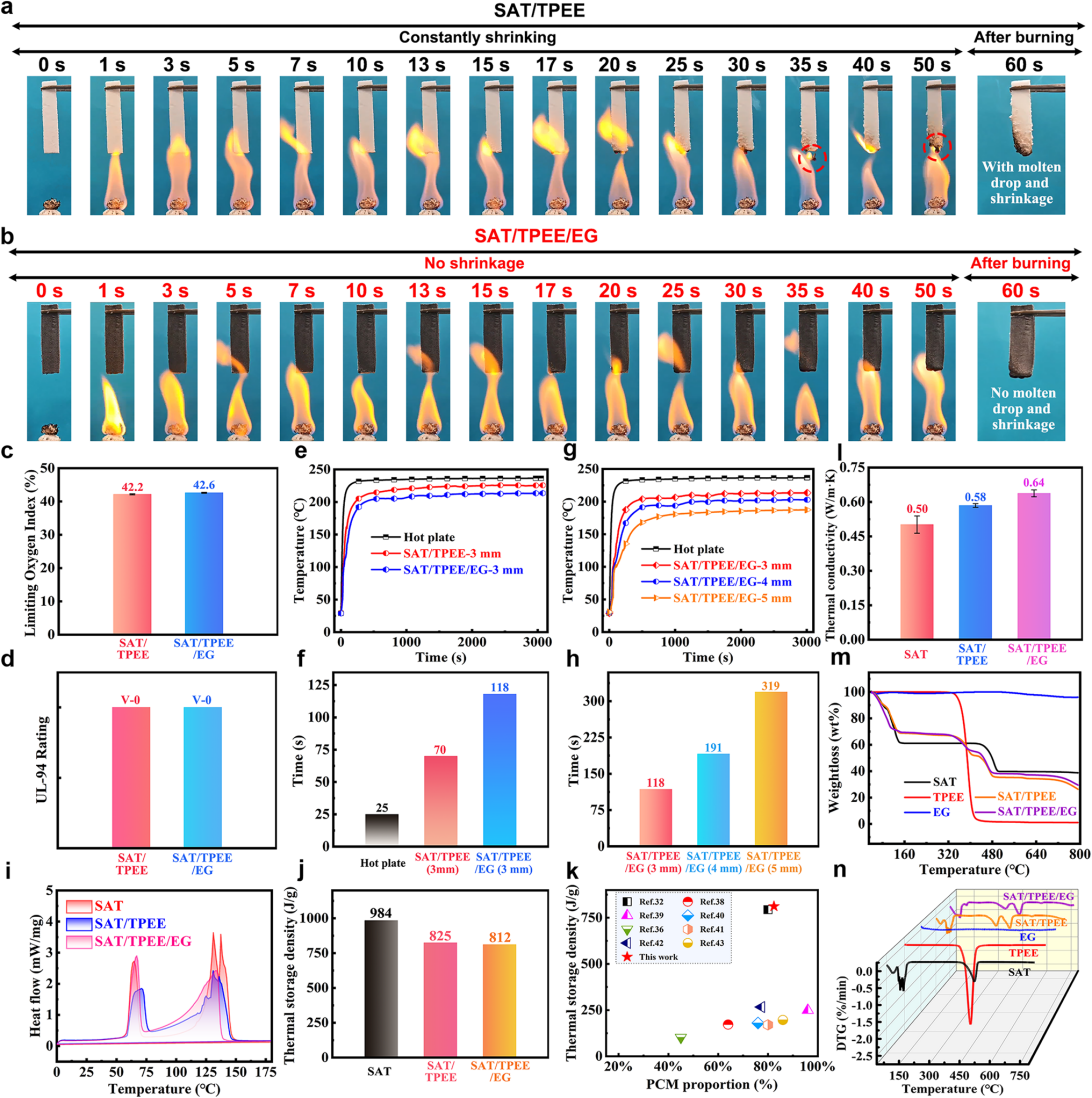
**a** and **b** Photographs showing the combustion process of SAT/TPEE and SAT/TPEE/EG over time. **c** LOI and **d** UL-94 ratings of SAT/TPEE and SAT/TPEE/EG. **e** and **f** Thermal insulation performance test of SAT/TPEE-3 mm and SAT/TPEE/EG-3 mm, and the time required for them to reach 150 °C. **g** and **h** Thermal insulation performance test of SAT/TPEE/EG with different thicknesses, and the time required for them to reach 150 °C. **i-j** DSC curves of SAT, SAT/TPEE, and SAT/TPEE/EG, and a comparison of their corresponding thermal storage density. **k** Comparison of the thermal storage density of CPCMs from different studies. **l** Thermal conductivity, **m** and **n** TG and DTG curves of SAT, TPEE, EG, SAT/TPEE, and SAT/TPEE/EG

To evaluate the flame retardancy of the CPCMs, we documented the combustion process of samples exposed to an alcohol lamp over time, as shown in Fig. 5a, b. It was observed that SAT/TPEE gradually shrinks, accompanied by dripping of molten material. In contrast, SAT/TPEE/EG does not exhibit shrinkage or dripping during combustion, indicating superior flame retardancy. Further flame retardancy assessments were conducted through LOI and vertical burning tests, with the results displayed in Fig. 5c, d. Both materials exhibit high LOI values, classifying them as flame-resistant, and both achieved the highest UL-94 V-0 rating, indicating excellent flame retardancy. However, based on the alcohol lamp combustion test, SAT/TPEE/EG demonstrates superior flame retardancy and fire resistance, primarily due to the dual encapsulation strategy.

Thermal insulation performance is also crucial for the practical application of CPCMs, so we evaluated their insulation properties (Fig. 5e-h). SAT/TPEE/EG has better thermal insulation performance, with SAT/TPEE reaching 150 °C in 70 s, while SAT/TPEE/EG takes 118 s. We also tested the thermal insulation performance of SAT/TPEE/EG at different thicknesses, revealing that insulation performance improves with increasing thickness. Considering that thermal protection materials should not be too thick, we determined that a thickness of 5 mm is optimal.

In evaluating the thermal protection capability of PCM systems, heat storage density plays an important role. Thus, we performed DSC tests on several PCMs and analyzed the results in Fig. 5i, j as well as Table 1. It can be seen that there are two peaks of SAT/TPEE/EG in the intervals of about 50–75 °C as well as 75–150 °C, which correspond to latent heat storage and thermochemical heat storage, respectively, and these two phases correspond to the loss of one water of crystallization and two water of crystallization of SAT, respectively. The enthalpy of phase change of CPCM was calculated to be 211.5 J g^−1^, the enthalpy of thermochemical decomposition to be 600.4 J g^−1^, and the total heat storage density to be 811.9 J g^−1^, respectively, which are higher than those of similar CPCMs reported [32], [36], [38], [39], [40], [41, [42], [43] so far (Fig. 5k and Table S5). The above results suggest that SAT/TPE/EG can absorb a large amount of heat in this temperature range, thus acting as an ‘energy storage’ for Ge/SA composite aerogels.

**Table 1:** Heat storage performance of different CPCMs

Sample	Heat storage temperature (°C)	Phase change enthalpy (J g^−1^)	Chemical decomposition enthalpy (J g^−1^)	Thermal storage density (J g^−1^)
SAT	64.5/131.7/137.3	253.2 ± 2.1	731.1 ± 2.7	984.3
SAT/TPEE	71.1/130.7	215.0 ± 0.7	609.6 ± 1.3	824.6
SAT/TPEE/EG	66.7/132.9	211.5 ± 0.7	600.4 ± 2.3	811.9

We also measured the thermal conductivity of SAT, SAT/TPEE, and SAT/TPEE/EG, with the results shown in Fig. 5l. It was found that the addition of EG slightly increased the overall thermal conductivity, raising it from 0.58 to 0.64 W m^−1^ K^−1^, which allows for quicker absorption of the accumulated heat in the composite aerogel. The thermal stability of CPCM is another important criterion for evaluating its application potential, so we conducted thermogravimetric analysis. As shown in Fig. 5m, n, the weight loss observed in SAT/TPEE/EG around 50–75 and 75–150 °C corresponds to the loss of one and two water molecules of crystallization from SAT, respectively.

### Ge/SA Composite Aerogel Coupled with STE CPCM

**Fig. 6:**
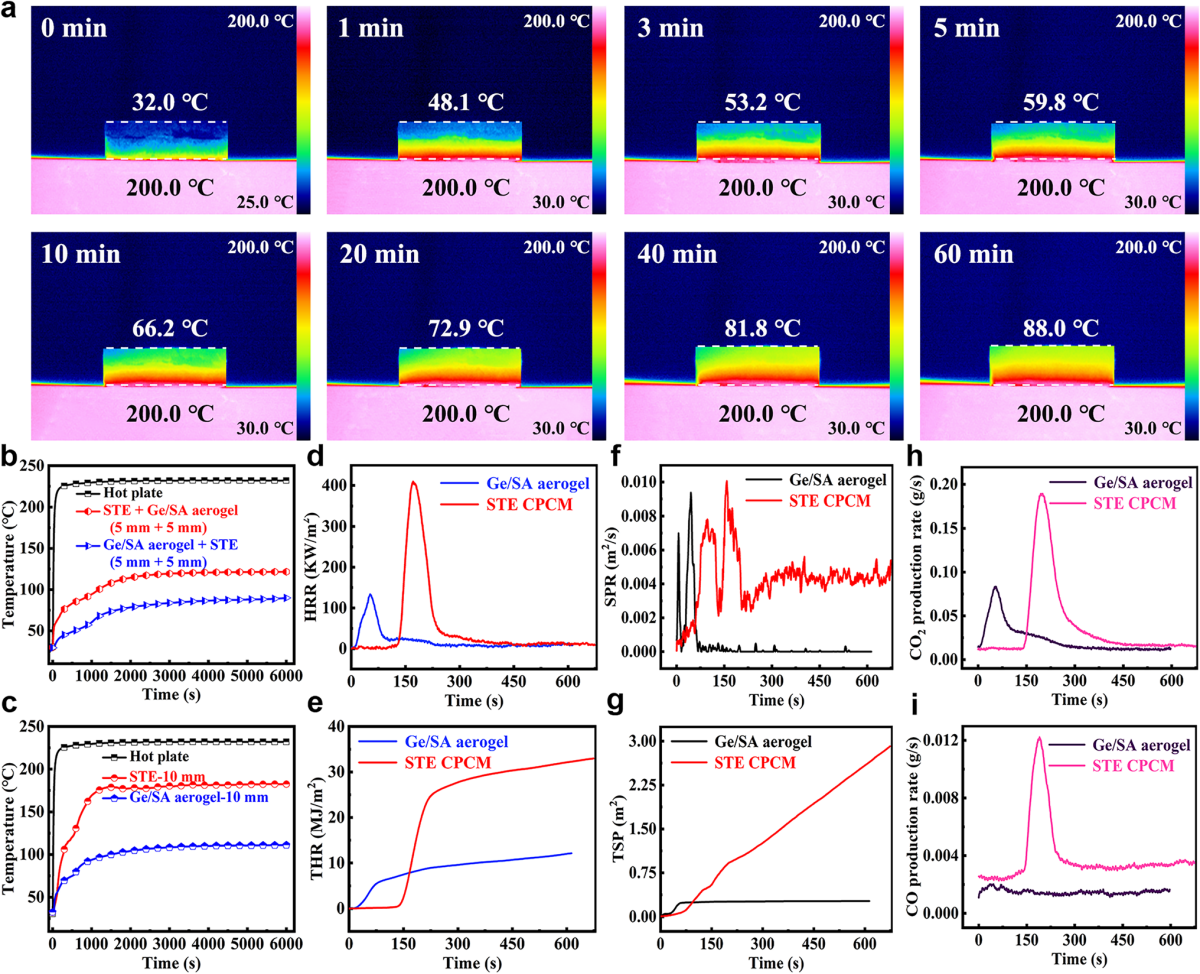
**a** Infrared thermal images of the Ge/SA-STE composite material at different temperatures. **b** and **c** Thermal insulation performance test of different samples on a heating plate and their corresponding temperature curves over time. Cone calorimetry test curves of Ge/SA composite aerogel and STE samples: **d** Heat Release Rate (HRR). **e** Total Heat Release (THR). **f** Smoke Production Rate (SPR). **g** Total Smoke Production (TSP). **h** CO_2_ release rate curve. **i** CO release rate curve

As mentioned in the introduction, in order to maximize the duration of thermal insulation and the efficiency of the thermal protection material, we have innovatively combined Ge/SA composite aerogel with SAT/TPPE/EG through the use of a binder to maximize the thermal insulation properties of the composite material. To determine the optimal combination method, we tested the thermal insulation performance of the Ge/SA-STE formed when Ge/SA aerogel was on top and when SAT/TPEE/EG was on top (Fig. 6b). When the Ge/SA aerogel is positioned closer to the heat source, the thermal insulation performance is superior, keeping the temperature below 90 °C. Additionally, we measured the thermal insulation performance of STE and Ge/SA aerogel at the same thickness (Fig. 6c). It was found that the final top temperature of STE was around 182 °C, while that of Ge/SA was 111 °C. Both are inferior to the thermal insulation performance of the coupled materials at the same thickness, demonstrating the superiority of our coupled material. To more intuitively evaluate the thermal insulation performance of Ge/SA-STE, we placed it on a 200 °C heating plate and used infrared thermal imaging to capture its temperature over time. The results, shown in Fig. 6a, reveal that Ge/SA-STE exhibits excellent thermal insulation performance. Even at a high temperature of 200 °C, the top surface temperature after 60 min was approximately 88 °C.

In order to further verify its thermal protection performance, we have coupled material tightly on the surface of the Li-ion battery, Li-ion battery TR test, as a comparison, but also on the Li-ion battery without coupling material TR test (TR test schematic shown in Fig. S12), the results are shown in Figs. S13 and S14. It can be seen that in the blank control group of Li-ion battery without coupling material, the TR of Li-ion battery occurs at 24 min 8 s, and at the same time, a large number of sparks erupt from the battery, and its temperature rises to 223.1 °C, and then the temperature begins to fall gradually, and the TR of Li-ion battery ends at about 25 min 2 s. It can be seen that Li-ion batteries in the TR, a short period of time will rise suddenly, a large number of sparks, very dangerous, which shows the importance of thermal protection materials. As for the group of Li-ion batteries with thermal protection materials, it can be seen from the figure that the TR of Li-ion batteries began to occur in 26 min 30 s, at which time the temperature on the surface of the Li-ion batteries rose to 227 °C; at this time, the temperature on the other side of the thermal protection materials was only 41.7 °C, and after a long time interval of 787 s, it slowly rose to the highest temperature of 59.8 °C, and then fell. This shows that thermal protection materials can delay the occurrence of TR to a certain extent, but also in a certain period of time to delay the transfer and spread of heat, to buy more time for emergency treatment.

Based on the above results, we propose a possible thermal insulation mechanism for the Ge/SA-STE coupled thermal protection material, as shown in Fig. S15. The Ge/SA aerogel acts as the first "barrier" of thermal protection, primarily serving as an insulator. Its excellent thermal insulation performance can be attributed to several factors: First, the Ge/SA aerogel has a very high porosity (approximately 90%), meaning that it contains a large amount of air, which has a much lower thermal conductivity than solid materials, resulting in low thermal conductivity for the aerogel and effectively preventing heat transfer. Second, the aerogel contains numerous micron-sized pores that restrict the free movement of gas molecules, reducing convective heat transfer between air particles. Additionally, the curved pathways of the Ge/SA aerogel’s nanomesh structure hinder both gaseous and solid-state heat conduction. Lastly, the aerogel’s nearly infinite number of pore walls minimizes thermal radiation. These combined effects effectively block all heat transfer pathways, providing the Ge/SA aerogel with outstanding thermal insulation performance. However, despite its excellent thermal insulation properties, the aerogel faces the issue of thermal saturation. When thermal saturation occurs, the Ge/SA aerogel can no longer block heat transfer, and this is where the STE CPCM as the second "barrier" of thermal protection comes into play. The STE CPCM has a very high heat storage density and can absorb a large amount of heat in the temperature range of 50–150 °C. When the aerogel begins to experience thermal saturation, the STE material undergoes phase change and thermochemical decomposition, absorbing a significant amount of heat from the aerogel, thereby alleviating the thermal saturation and allowing the aerogel to continue functioning as a thermal insulator, delaying heat transfer. Moreover, the STE CPCM exhibits good thermally induced flexibility. When the temperature reaches around 50 °C, the STE becomes highly flexible, enabling it to fill the interface between the STE and the Ge/SA aerogel more effectively, reducing the contact thermal resistance. This allows the STE to absorb heat from the Ge/SA aerogel more quickly, further delaying thermal saturation and providing additional time for emergency response. This mechanism, where the STE CPCM continuously "recharges" the Ge/SA aerogel by absorbing and dissipating heat in a timely manner, endows the Ge/SA-STE coupled thermal protection material with superior thermal insulation performance, significantly delaying heat transfer.

To assess the potential harm of the composite material to human health during a fire, we used cone calorimetry to characterize the material. Since Ge/SA-STE is only physically bonded between the Ge/SA composite aerogel and STE, we tested the Ge/SA composite aerogel and SAT/TPEE/EG separately (Fig. 6d−i). Figure 6d, e shows that SAT/TPEE/EG is a flame-retardant CPCM, with HRR and THR values of 409.5 KW m^−2^ and 33.0 MJ m^−2^, respectively. The Ge/SA composite aerogel is also a flame-retardant material, with even lower HRR and THR values of 133.7 KW m^−2^ and 12.2 MJ m^−2^, respectively. These results indicate that both materials have excellent flame retardancy. Additionally, vertical burning tests showed that both materials achieved the highest V-0 rating for flame retardancy (Table S3).

The release of toxic or harmful gases, such as smoke, CO, and CO_2_, during combustion is one of the primary concerns as it can lead to asphyxiation and injury. Therefore, reducing the release of these gases is crucial for improving the safety of flame-retardant materials. Figure 6f, g shows that the TSP values of SAT/TPEE/EG and Ge/SA composite aerogel are 2.91 and 0.27 m^2^, respectively. Furthermore, as seen in Fig. 6h, i, the CO and CO_2_ emissions from SAT/TPEE/EG and Ge/SA composite aerogel are minimal, indicating that the composite materials have excellent smoke suppression capabilities.

**Fig. 7:**
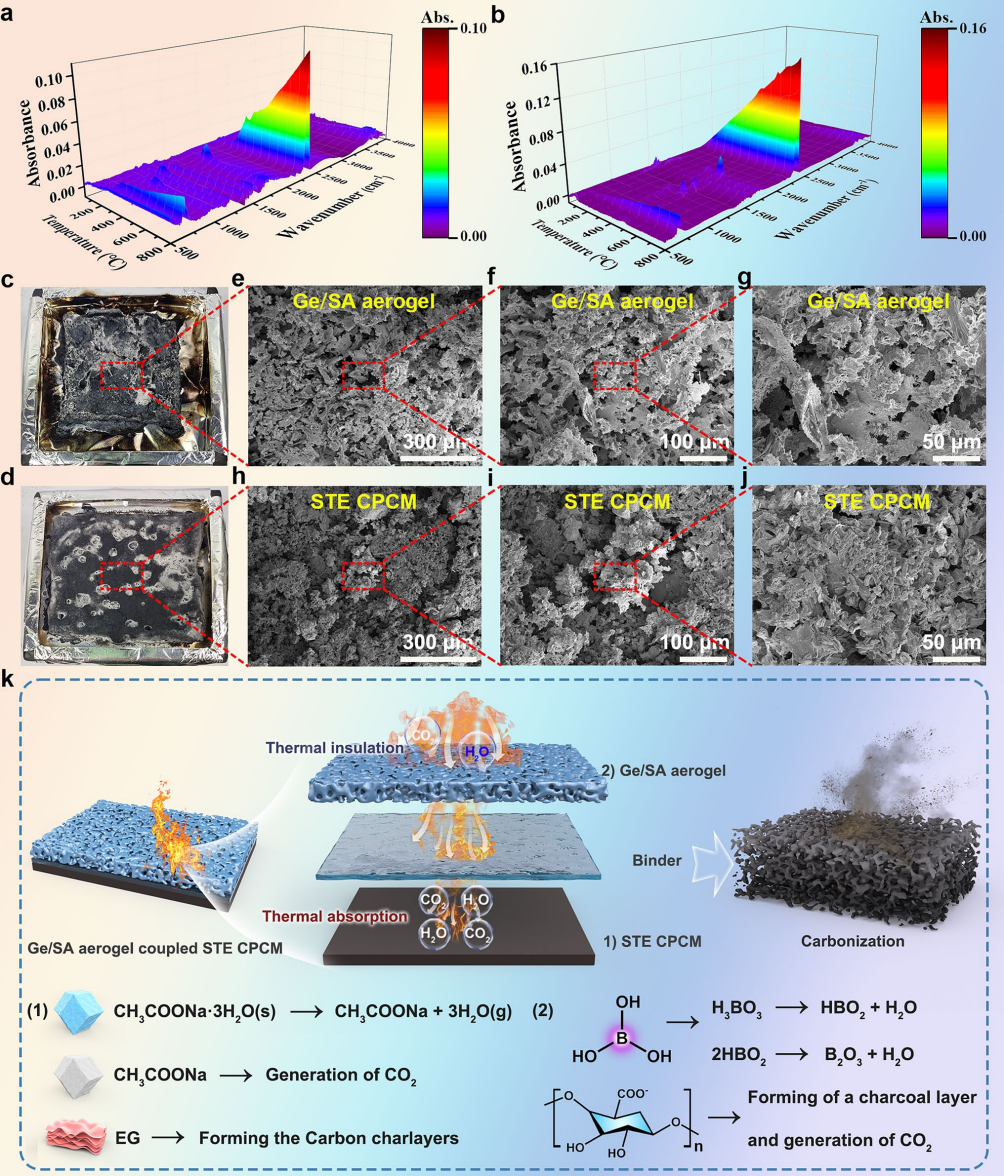
**a** 3D TG-IR spectrum of the Ge/SA composite aerogel and **b** STE composite material. **c** and **d** Char residues of Ge/SA composite aerogel and STE CPCM after cone calorimetry testing, along with their corresponding **e**–**j** SEM images. **k** Proposed flame-retardant mechanism of the Ge/SA-STE composite material

To investigate the flame-retardant mechanism of the Ge/SA composite aerogel and STE composite material, we conducted TG-IR characterization (Figs. 7a, b and S16, S17). As seen in Figs. 7a and S16, when the temperature increases from 100 to 800 °C, the absorption peak attributed to H_2_O at 3600–3800 cm^−1^ in the Ge/SA aerogel gradually intensifies. This is primarily because SA and BA decompose under high temperatures, releasing water vapor. Simultaneously, when the temperature exceeds 100 °C, the characteristic absorption peak of CO_2_ at 2300–2400 cm^−1^ becomes more pronounced with rising temperature, due to the breakdown and decarboxylation of SA, which generates a significant amount of CO_2_ [44]. The characteristic peaks at 1700–1830 and 1200 cm^−1^ correspond to the stretching vibrations of C = O and C-O, respectively, indicating the formation of small volatile molecules such as aldehydes, alcohols, and esters when SA is exposed to high temperatures. When the temperature exceeds 200 °C, the characteristic peak at 670 cm^−1^ gradually intensifies, attributed to the stretching vibration of B−O−B, which is a result of the thermal decomposition of BA into B_2_O_3_ [45]. In Figs. 7b and S17, the thermal decomposition products of STE show similarities to those of the Ge/SA composite aerogel, but with some differences. When the temperature exceeds 100 °C, an absorption peak of H_2_O at 3600–3800 cm^−1^ gradually appears in STE, which is caused by the loss of crystallization water from SAT. Additionally, the characteristic absorption peak of CO_2_ at 2300–2400 cm^−1^ increases with temperature, due to the decomposition of sodium acetate generating large amounts of CO_2_. The characteristic peaks at 1700–1830, 1368, and 1212 cm^−1^ correspond to the stretching vibrations of C = O, -CH_3_ deformation vibration, and C−O, respectively, indicating that TPEE produces small volatile molecules such as aldehydes, esters, and alkanes at high temperatures. When the temperature exceeds 200 °C, the characteristic peak at 640–670 cm^−1^ intensifies, which is attributed to the stretching vibration of C = C in EG. These results indicate that during the thermal decomposition process, the Ge/SA-STE composite material releases non-flammable gases such as water vapor and CO_2_, which dilute the concentration of O_2_ in the air, thereby enhancing the flame-retardant properties of the composite material.

To further elucidate the condensed-phase flame retardant mechanism of the Ge/SA-STE composite material, we performed characterization analyses on the char residues left after cone calorimetry testing. The results are shown in Figs. 7c-j and S18, S19. As observed in the figures, the residue of the Ge/SA composite aerogel exhibits numerous pores on its surface, likely due to the release of gases during combustion. In contrast, the residue of the STE composite material has a denser surface, which can act as an insulating barrier to prevent mass and heat exchange. Additionally, we conducted XRD characterization of the residues from the Ge/SA composite aerogel and STE samples (Fig. S18). The XRD results indicate that the characteristic peaks of the Ge/SA composite aerogel residue are extremely weak, suggesting it is an amorphous material rather than a crystalline structure. Meanwhile, the STE residue shows strong characteristic diffraction peaks, primarily composed of graphitized char and sodium acetate. We also performed FT-IR characterization (Fig. S19). Both materials exhibit absorption peaks at 1426 and 864 cm^−1^, corresponding to the symmetric stretching vibration of C = O and the symmetric bending vibration of C–O–C, respectively. These peaks may be attributed to the residual alginate in the Ge/SA composite aerogel, while in the STE, they result from the remaining sodium acetate after the loss of three water molecules from SAT.

Based on the analysis of the gaseous and solid-phase components, we proposed a potential flame-retardant mechanism for the Ge/SA-STE composite material, as illustrated in Fig. 7k. The figure suggests that the composite material exhibits synergistic flame-retardant behavior in both the gas phase and the condensed phase. The flame-retardant mechanism of composite material can be attributed to the following aspects: (1) Dilution effect: During combustion, SAT, H_3_BO_3_, and SA release non-flammable gases (such as H_2_O and CO_2_), which dilute the concentration of flammable oxygen and combustible volatiles, creating an inert atmosphere that slows down the combustion in the flame zone; (2) Phase change effect: The SAT/TPEE/EG undergoes a phase change during combustion, absorbing a large amount of heat in the process, which delays the burning of the sample; (3) Physical barrier effect: During combustion, BA ultimately decomposes into B_2_O_3_, a high-temperature-resistant inorganic component that covers the char surface, forming a protective layer. This layer effectively reduces the diffusion rate of internal pyrolysis products, preventing the underlying substrate from further flame attack; (4) Charring effect: EG and SA rearrange under the influence of heat and oxygen to form a dense char layer. This dense char layer acts as a barrier, isolating heat and oxygen, thereby slowing the pyrolysis process and inhibiting flame propagation.

## Conclusions

In summary, we successfully developed a thermal protective material applicable to TR in Li-ion batteries. This composite material primarily consists of two components: One is a Ge/SA biomass-based composite aerogel with a multiple crosslinking network, and the other is a SAT/TPEE/EG CPCM with thermally induced flexibility. Inspired by power banks, we coupled the two through the binder, using the CPCM can be constantly for the aerogel ‘charging energy storage’, in time for the absorption of heat for the characteristics of this phenomenon, delay the occurrence of thermal saturation phenomenon, and maximize the coupling of composite materials to improve the length of the thermal insulation. This characteristic maximizes the delay of thermal saturation and enhances the thermal protection duration of the coupled composite material. The research results indicate that the Ge/SA aerogel exhibits excellent thermal insulation properties (with a temperature difference of approximately 120 °C at a thickness of 1 cm), good mechanical performance, and outstanding flame-retardant properties (V-0 rating). The prepared CPCM possesses a high heat storage density of up to 811.9 J g^−1^, along with good flame retardancy (V-0 rating) and thermally induced flexibility (bendable above 40 °C). Furthermore, the coupled composite material, Ge/SA-STE, demonstrates high thermal insulation properties, maintaining a temperature of around 89 °C at a high temperature of 230 °C, making it highly suitable for applications in the field of Li-ion battery TR.

## Supplementary Information

Below is the link to the electronic supplementary material.

Supplementary file1 (DOCX 2921 kb)

## Abbreviations

∆T: Temperature difference
°C: Degree celsius
C: Battery charge and discharge capacity ratio
s: Seconds
min: Minutes
g: Grams
h: Hours
μL: Microliters
mm: Millimetres
Hz: Hertz
*R*: Thermal resistance (°C W^−^^1^)
∆*H*: Enthalpy value (J g^−^^1^)
*T*: Temperature (°C)
*t*: Time (min)

## List of symbols

TR: Thermal runaway
CPCM: Composite phase change material
SAT: Sodium acetate trihydrate
SA: Sodium alginate
Li-ion: Lithium-ion
PCMs: Phase change materials
Ge: Gelatin
GLU: Glutaraldehyde
BA: Boric acid
TPEE: Thermoplastic polyester elastomer
EG: Expanded graphite
LOI: Limit oxygen index

## Subscripts

max: Maximum
min: Minimum
T_x_: Thermocouple

## Greek symbols

λ: Thermal conductivity (W m^−^^1^ K^−^^1^)
