# Supplementary material for: Enhancing Thermal Protection in Lithium Batteries with Power Bank-Inspired Multi-Network Aerogel and Thermally Induced Flexible Composite Phase Change Material
Source: Nanomicro Lett. 2025 Feb 26;17:166. doi: 10.1007/s40820-024-01593-0 (PMC11865416; doi:10.1007/s40820-024-01593-0)
Supplement: Supplementary file 1 — Supplementary file1 (DOCX 2921 kb) [file 40820_2024_1593_MOESM1_ESM.docx]

Supporting Information for

**Enhancing Thermal Protection in Lithium Batteries with Power Bank-Inspired Multi-Network Aerogel and Thermally Induced Flexible Composite Phase Change Material**

Zaichao Li^1^, Feng Cao^1^, Yuang Zhang^1,^ *, Shufen Zhang^1^, Bingtao Tang^1,^ *

^1^State Key Laboratory of Fine Chemicals, Frontiers Science Center for Smart Materials Oriented Chemical Engineering, Dalian University of Technology, Dalian 116024, P. R. China

*Corresponding authors. E-mail: [zhangyuang@dlut.edu.cn](mailto:zhangyuang@dlut.edu.cn) (Yuang Zhang); [tangbt@dlut.edu.cn](mailto:tangbt@dlut.edu.cn) (Bingtao Tang)

**S1 Experimental Sections**

**S1.1 Materials**

Ge (analytical grade) and a 50% GLU solution were procured from Shanghai Aladdin Biochemical Technology Co., Ltd. SA with a viscosity ranging between 7000-10000 mPa·s, BA (>99.5%), SAT (99%), and a 0.2 mol/L acetic acid solution were sourced from Shanghai Macklin Biochemical Technology Co., Ltd. The TPEE (SK G130D) was supplied by China Konai New Materials Co., Ltd., while EG (50 mesh) was obtained from Qingdao Tengshengda Carbon Machinery Co., Ltd. The adhesive (LN W1350) was provided by China Lilin Co., Ltd., and dichloromethane was acquired from China National Pharmaceutical Group Co., Ltd. Unless otherwise noted, all reagents used in this study were of analytical grade.

**S1.2 Characterization**

The surface morphology and microstructure of the samples were analyzed using scanning electron microscopy (SEM). X-ray powder diffraction (XRD) analysis was performed with a Rigaku Smart Lab X-ray diffractometer to determine the crystal phase structure. Fourier transform infrared spectroscopy (FT-IR, NICOLETT, USA) was employed to investigate the chemical structure of the samples. The pore structure properties were characterized using a fully automated multi-station micropore physical adsorption analyzer (3FLEX, Micromeritics, USA) and a mercury intrusion porosimeter (MicroActive AutoPore V 9600). The mechanical properties were assessed using a universal testing machine (INSTRON 5982) at a compression rate of 5 mm/min.

The flame retardancy of the samples was evaluated using the limiting oxygen index (LOI) method (FTT0077, UK), where a higher oxygen requirement for sustained combustion indicates better flame retardancy. A vertical burning test (FTT0082, UK) was employed to assess the flame retardant rating of the samples. Specimens measuring 125 × 13 × 10 mm^3^ were vertically aligned (15 parallel samples). The materials were ignited twice, each for a duration of 10 s. If the flames extinguished within 10 s both times without any burning material dropping, the sample was rated as V-0. If the total flame duration did not exceed 30 s and the dripping material did not ignite the cotton placed below, the sample was rated as V-1. If the total flame duration did not exceed 30 s but the dripping material ignited the cotton, the sample was rated as V-2. Heat release rates, smoke production, and toxic gas emissions were measured using cone calorimetry (ISO5660, 35 KW). Thermal stability was determined through thermogravimetric analysis (TGA, TG 209, TA), with testing conducted from room temperature to 800 ℃ under a nitrogen atmosphere at a heating rate of 10 ℃/min. Decomposition products were analyzed using combined TG-FTIR (TGA8000-Frontier-Clarus SQ8T, 10 ℃/min).

Differential scanning calorimetry (DSC, DSC Q20) and reaction calorimetry were employed to measure the phase transition temperature and enthalpy of the composite PCMs over a range of 0 to 180 ℃ under a nitrogen atmosphere at a heating rate of 5 ℃/min. Thermo-mechanical properties were measured using a torque rheometer (Thermo Fisher Mars40) with a heating rate of 5 ℃/min, a frequency of 1 Hz, and a scanning temperature range of 10-70 ℃. The thermal conductivity of the composites was measured using a thermal conductivity meter (Hot Disk TPS 2500S).

An infrared thermal imager (FLUKE, USA) was used to capture the temperature distribution of the samples, and digital photographs were taken using a digital camera (Nikon, Japan). The shape stability of the samples was tested through time-lapse photography. The leakage test procedure was as follows: two different samples, each measuring 50×50×8 mm^3^, were placed on a heating plate at 80 ℃. A digital camera and infrared thermal imager were used to track and capture the appearance of the samples in real-time as the temperature changed. Digital photos and infrared thermal images were taken at 0, 5, 10, and 30 min. To better observe any leaked liquid, the heating plate was tilted at a certain angle (with hard objects placed underneath the plate to achieve the tilt). The specific test setup is shown in Fig. S10.

The leakage rate of the samples was tested using the following method: First, the original mass of the sample ($Q_{original}$) was measured. The sample was then heated on an 80 ℃ heating plate, and at specific intervals, the remaining solid mass ($Q_{n}$) was measured. The leakage rate was calculated using Eq. (S1) [S1]:

$$\begin{aligned} R_{LR}=(1-\frac{Q_{n}}{Q_{original}}) \times100\%\#\left( S1 \right) \end{aligned}$$

The contact thermal resistance of the CPCM was measured using a steady-state thermal method. A ceramic heating block (35×20×5 mm) was placed on the CPCM, with the other surfaces insulated using thermal insulation cotton to ensure unidirectional heat transfer through the CPCM. Thermocouples *T_1_*, *T_2_*, and *T_3_* were used to monitor real-time temperatures at the center of the Li-ion battery surface, inside the CPCM, and on the CPCM surface, respectively. Assuming uniform thermal conductivity, the temperature at the CPCM-ceramic heating block interface (*T_PCM_*) was calculated using thermal equilibrium equations (2 and 3 below). The interface temperature (*T_PCM_*) and thermal resistance (*R*) were determined as follows [S2]:

$$\begin{aligned} T_{PCM}-T_{2}=T_{2}-T_{3} \#\left( S2 \right) \end{aligned}$$

$$\begin{aligned} R=\frac{T_{1}-T_{PCM}}{q}\#\left( S3 \right) \end{aligned}$$

Where *T_PCM_* represents the interface temperature, and *q* denotes the heating power supplied by the DC power source.

**S2 Supplementary Figures**


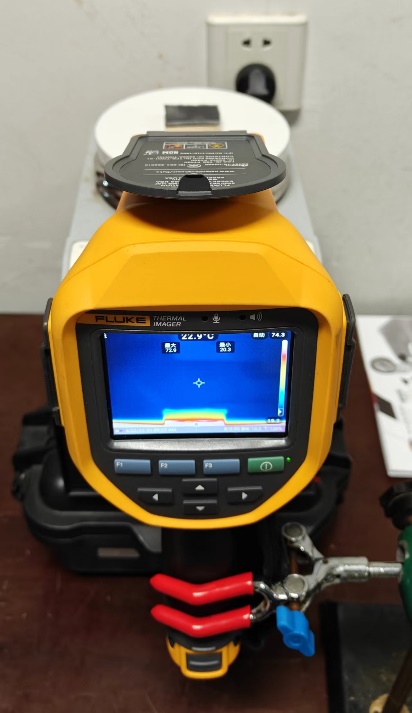


**Fig. S1** The schematic diagram of the thermal insulation test of the sample to be tested


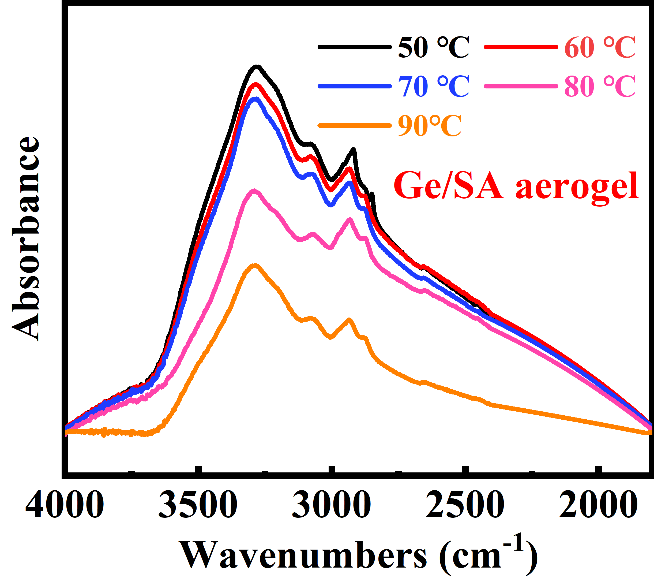


**Fig. S2** Infrared spectra of Ge/SA aerogel at different temperatures


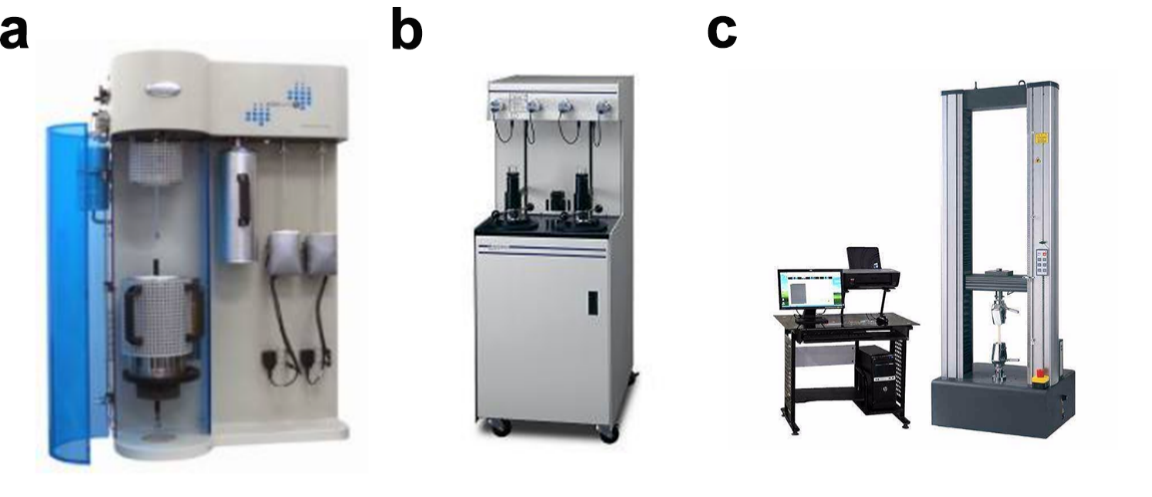


**Fig. S3 a** physical adsorption of nitrogen, **b** mercury porosimeter and **c** physical map of compression test device


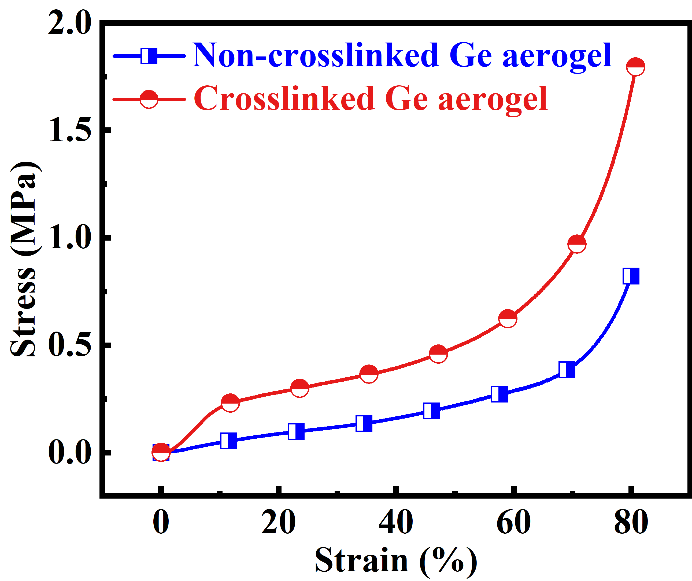


**Fig. S4** Compressive stress-strain curves of non-crosslinked Ge aerogel and crosslinked Ge aerogel


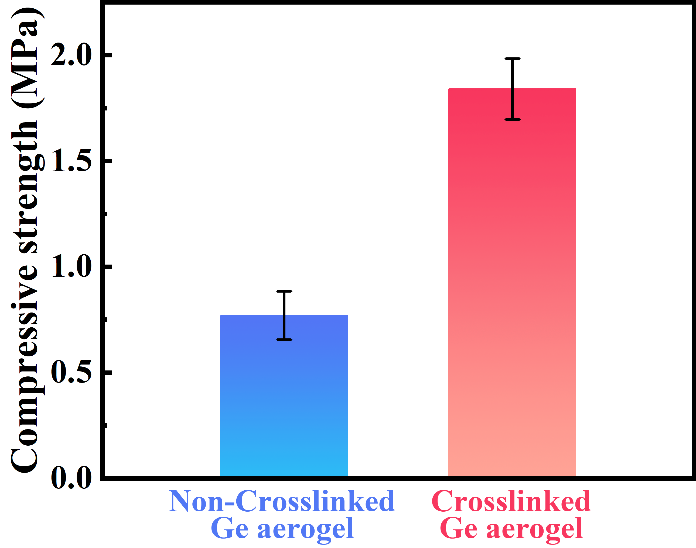


**Fig. S5** Comparison of compressive strength of non-crosslinked Ge aerogel and crosslinked Ge aerogel


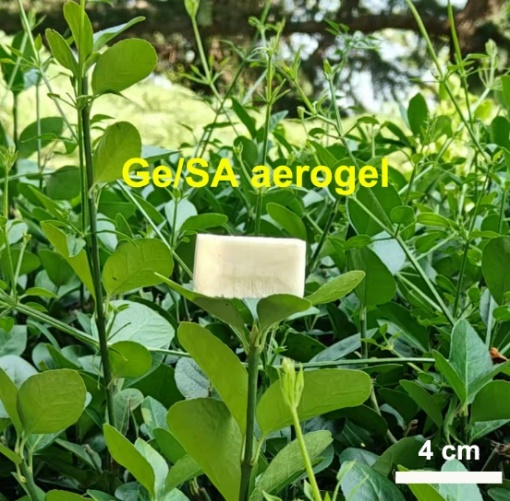


**Fig. S6** Digital photo of Ge/SA aerogel


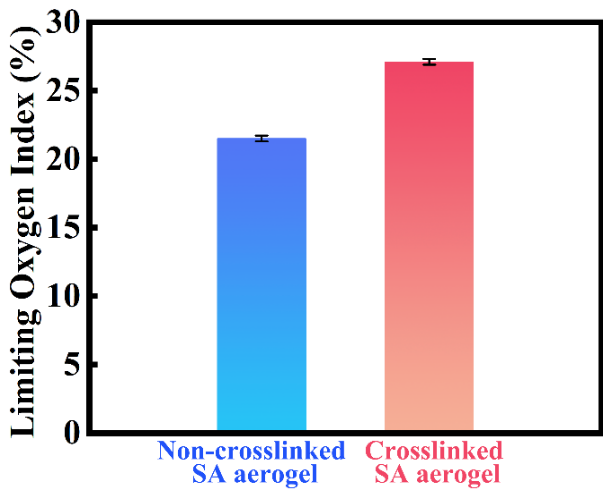


**Fig. S7** Comparison of limiting oxygen index of non-crosslinked SA aerogel and SA aerogel crosslinked with boric acid


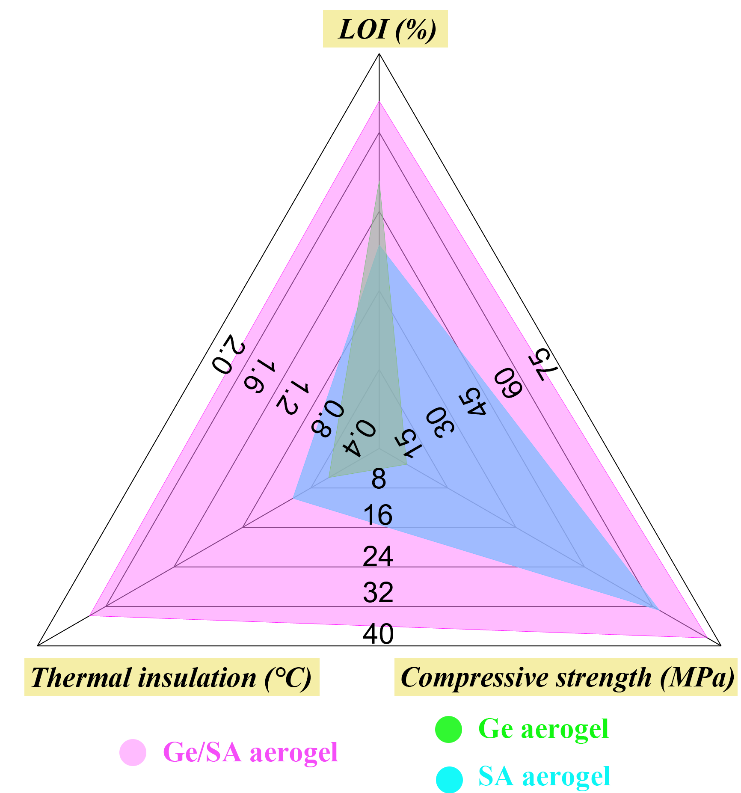


**Fig. S8** Comparison of radar chart performance between different aerogels


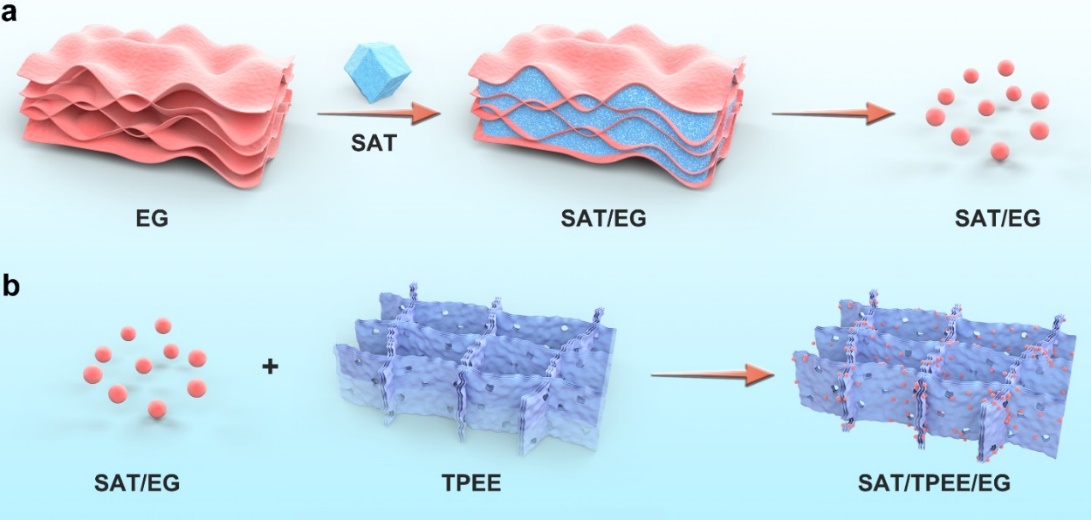


**Fig. S9** The schematic diagram of the preparation of SAT/TPEE/EG CPCM


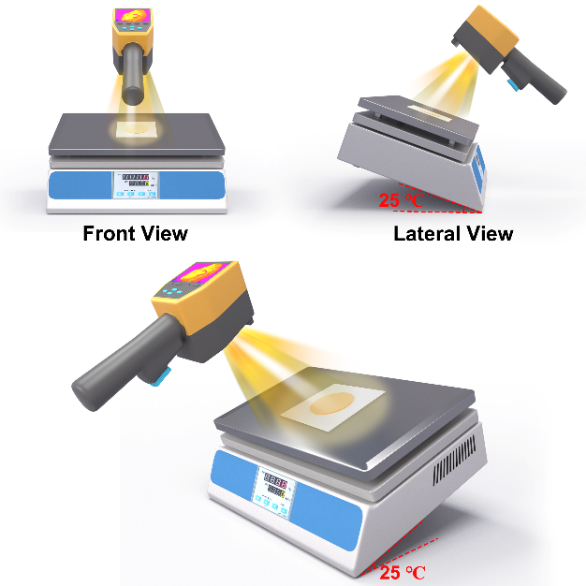


**Fig. S10** The schematic diagram of leakage test


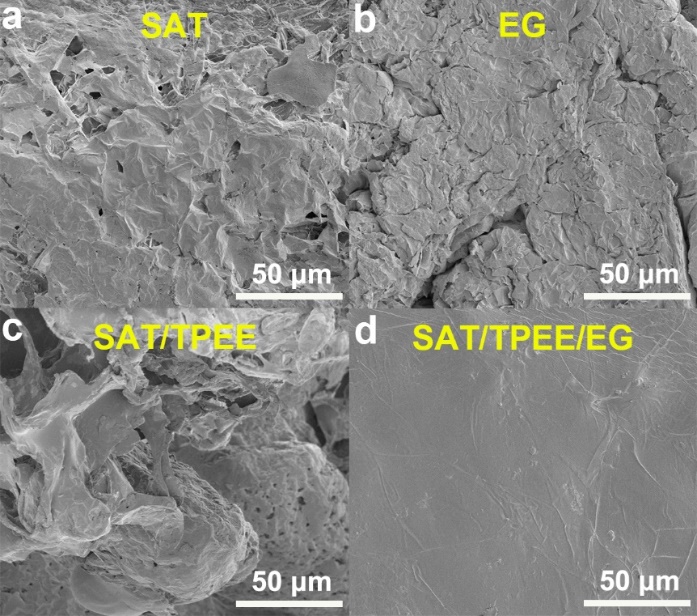


**Fig. S11 a-d** SEM images of SAT, TPEE, EG and SAT/TPEE/EG at 50 μm scale


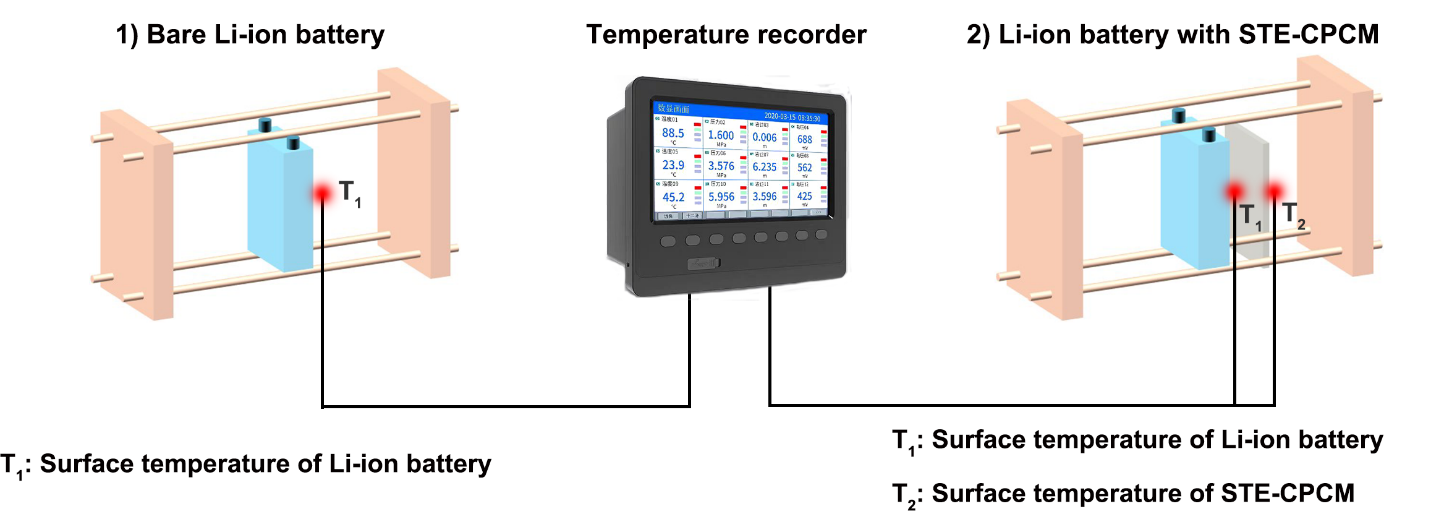


**Fig. S12** Chart of thermocouple arrangement for thermal runaway test of Li-ion battery


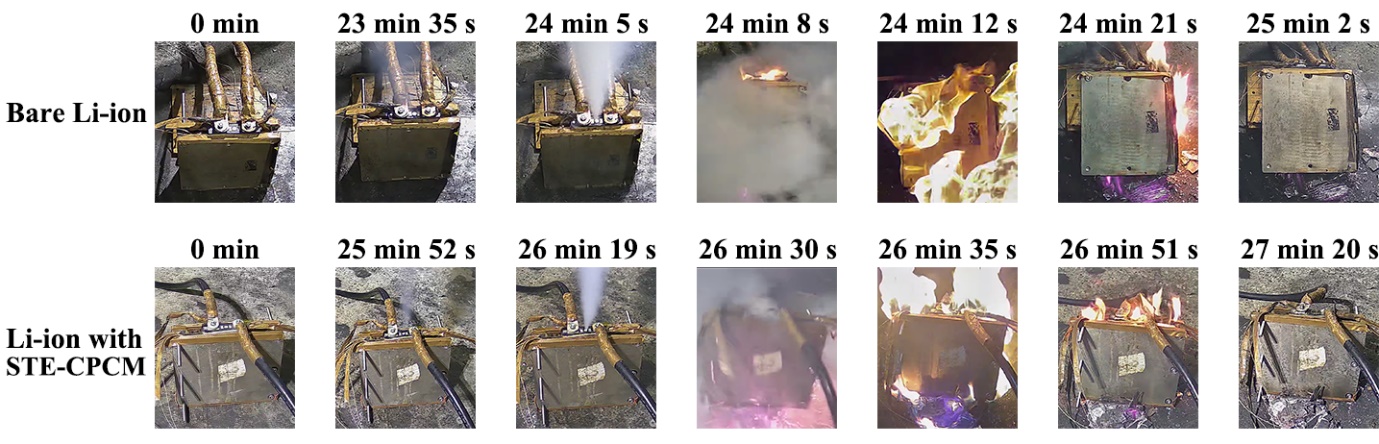


**Fig. S13** Digital photos of lithium batteries with and without thermal protection materials at different times when thermal runaway occurs


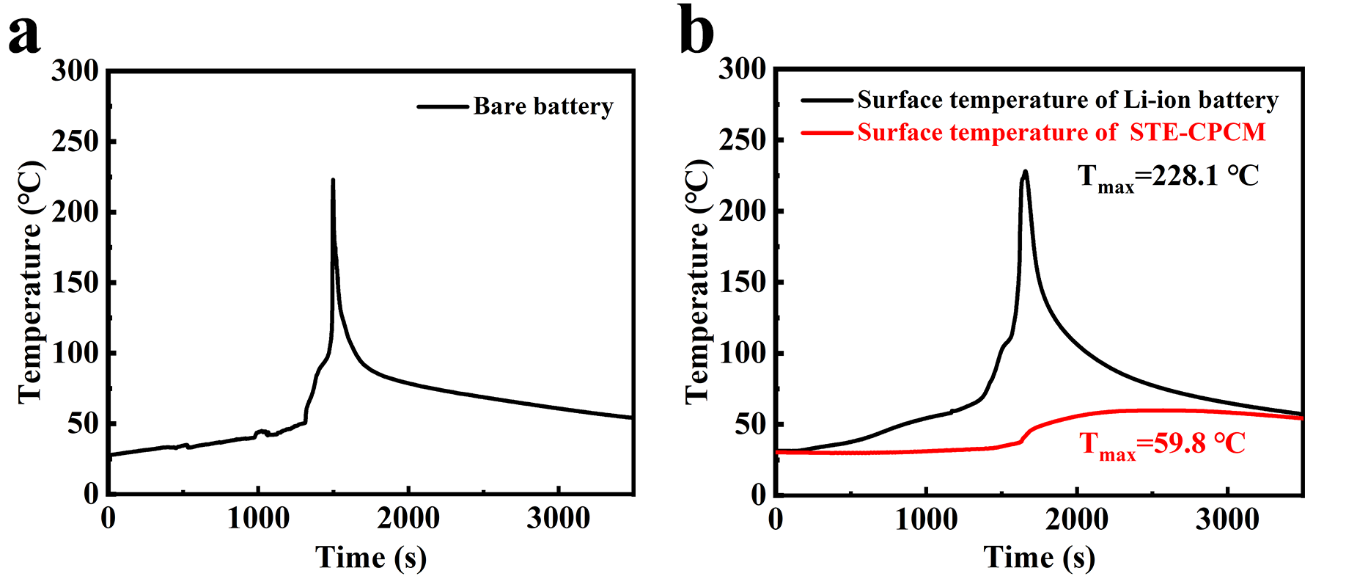


**Fig. S14** Temperature change curve of **a** bare battery and **b** Li-ion battery with thermal protection material during thermal runaway


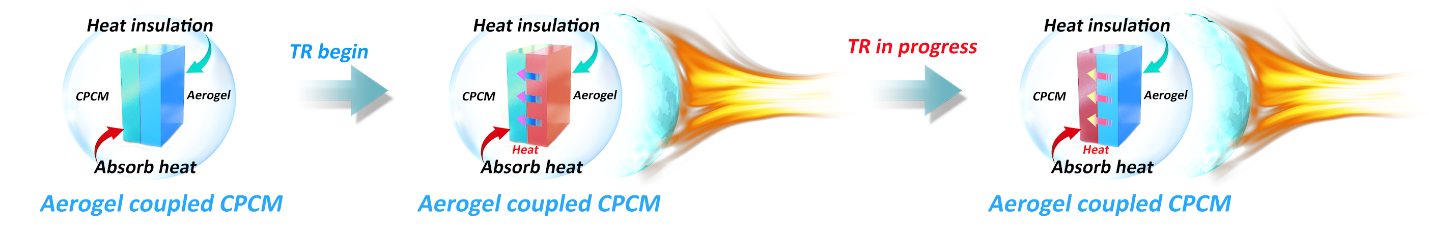


**Fig. S15** Possible thermal insulation mechanism of Ge/SA-STE coupled thermal protection materials


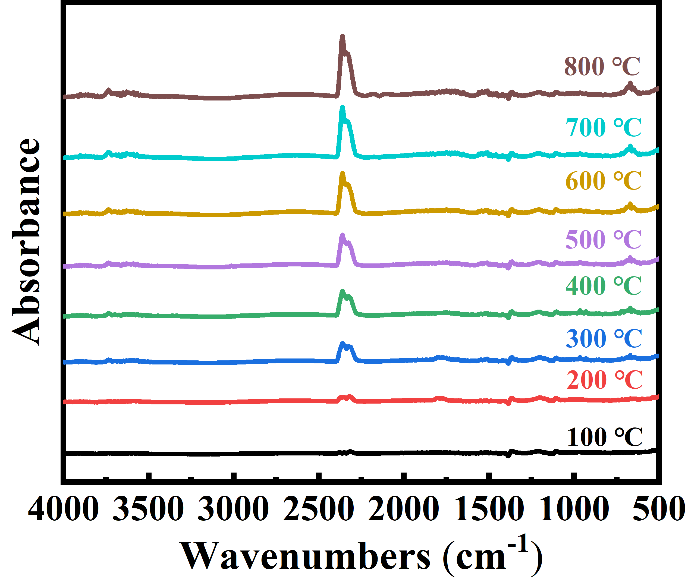


**Fig. S16** FT-IR spectra of pyrolysis products of Ge/SA composite aerogel at different temperatures


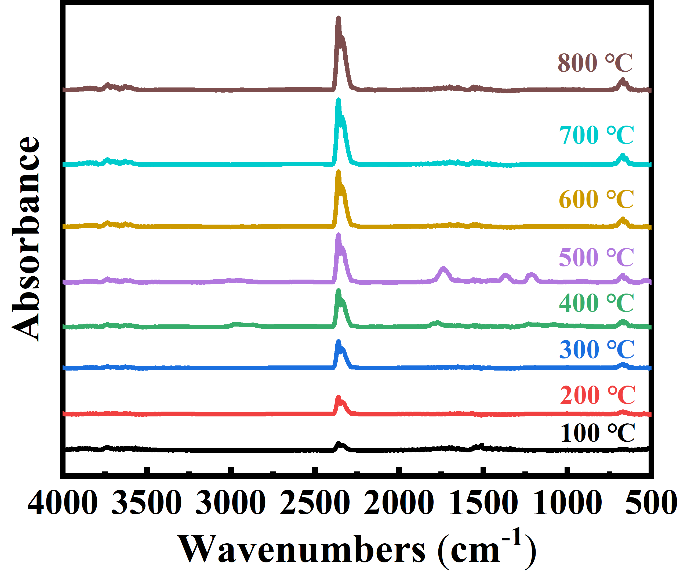


**Fig. S17** FT-IR spectra of pyrolysis products of SAT/TPEE/EG at different temperatures


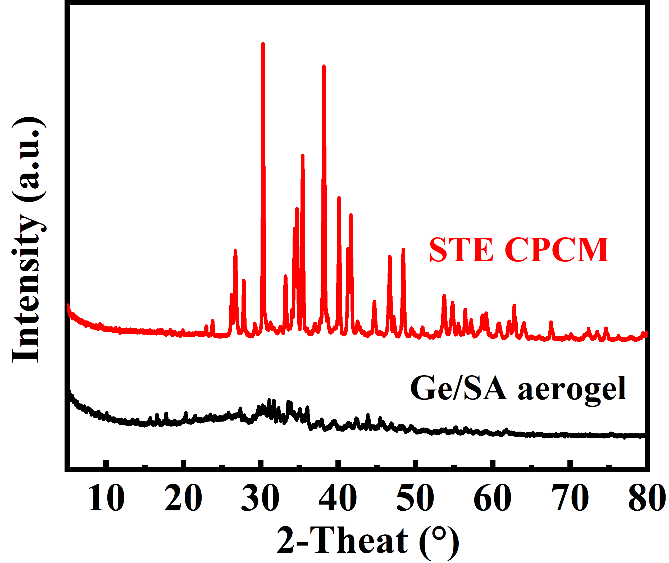


**Fig. S18** XRD patterns of Ge/SA composite aerogel and SAT/TPEE/EG carbon residue were obtained


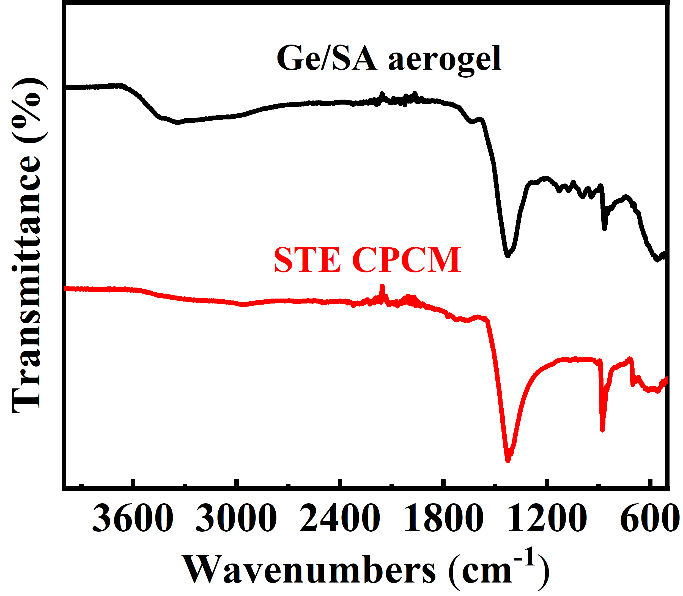


**Fig. S19** FT-IR patterns of Ge/SA composite aerogel and SAT/TPEE/EG carbon residue were obtained

**S3 Supplementary Tables**

**Table S1** The content of each component in the sample

| Sample | SAT/g | TPEE/g | EG/g |
| --- | --- | --- | --- |
| SAT/TPEE/EG (5 mm) | 22.0 | 4.0 | 0.7 |

**Table S2** Technical parameters of the battery module

| Property | Parameter |
| --- | --- |
| Capacity (Ah) | 30.0 |
| Nominal voltage (V) | 3.2 |
| Weight (g) | 640.0 |
| Height (mm) | 100.0 |
| Width (mm) | 140.0 |
| Thickness (mm) | 20.5 |
| Electrolyte material | Lithium hexafluorophosphate + Carbonate solvent + Ethylene carbonate |
| Cathode material | Lithium iron phosphate |
| Anode material | Graphite |

**Table S3** Rating of samples vertical combustion test

| Sample | UL-94 Rating |
| --- | --- |
| Ge/SA aerogel | V-0 |
| SAT/TPEE/EG | V-0 |
| Ge/SA-STE | V-0 |

**Table S4** Leakage results of the different PCMs

| Time  (min) | Weight variation (g/%) | | | |
| --- | --- | --- | --- | --- |
|  | SAT/TPEE | Leakage/% | SAT/TPEE/EG | Leakage/% |
| 0 | 1.790 | 0.000 | 1.844 | 0.000 |
| 10 | 1.725 | 3.631 | 1.844 | 0.000 |
| 20 | 1.675 | 6.425 | 1.844 | 0.000 |
| 30 | 1.628 | 9.050 | 1.844 | 0.000 |
| 60 | 1.534 | 14.302 | 1.844 | 0.000 |
| 90 | 1.413 | 21.061 | 1.844 | 0.000 |
| 120 | 1.292 | 27.821 | 1.844 | 0.000 |

**Table S5** Summaries of different CPCMs

| CPCM | Proportion | Heat storage temperature  (℃) | Thermal storage density  (J/g) | Refs. |
| --- | --- | --- | --- | --- |
| SAT/EG | 80.0%/20.0% | 58.5/106.5 | 793.4 | [S3] |
| SAT/bentonite/SC*^a^* | 64.0%/26.0%/10.0% | 57.5 | 171.9 | [S4] |
| SAT/SiC*^a^*/EG | 96.0%/2.0%/2.0% | 60.0 | 249.5 | [S5] |
| SAT/UREA/DHPD*^b^*/CMC*^c^*/CNTs*^d^*/EG | 76.0%/24.0% | 55.8 | 180.1 | [S6] |
| PA*^e^*/TPEE/EG | 45.0%/45.0%/10.0% | / | 102.0 | [S7] |
| PA/TPEE/SBS*^f^*/EG | 80.0%/5.0%/10.0%/5.0% | 55.0 | 170.1 | [S8] |
| *n*-eicosane/SAT/EG | 19.3%/77.3%/3.4% | 36.3/58.2 | 266.5 | [S9] |
| SAT-LiCl*^g^*-KCl*^h^*/EG | 86.0%/14.0% | 49.3 | 196.4 | [S10] |
| SAT/TPEE/EG | 82.4%/15.0%/2.6% | 66.7/132.9 | 811.9 | This work |

*^a^*SC/SiC: Silicon carbide. *^b^*DHPD: Disodium hydrogen phosphate dodecahydrate

*^c^*CMC: Sodium carboxymethylcellulose. *^d^*CNTs: Carbon nanotubes. *^e^*PA: Paraffin.

*^f^*SBS: Styrene-butadiene-styrene triblock copolymer. *^g^*LiCl: Lithium chloride

*^h^*KCl: Potassium chloride

# Supplementary References

1. W. Wu, W. Wu, S. Wang Form-stable and thermally induced flexible composite phase change material for thermal energy storage and thermal management applications. Appl. Energy **236**, 10–21 (2019). <https://doi.org/10.1016/j.apenergy.2018.11.071>
2. X. Liu, C. Wang, T. Wu, Z. Li, C. Wu A novel stable and flexible composite phase change materials for battery thermal management. Appl. Therm. Eng. **212**, 118510 (2022). <https://doi.org/10.1016/j.applthermaleng.2022.118510>
3. J. Cao, Z. Ling, S. Lin, Y. He, X. Fang et al., Thermochemical heat storage system for preventing battery thermal runaway propagation using sodium acetate trihydrate/expanded graphite. Chem. Eng. J. **433**, 133536 (2022). <https://doi.org/10.1016/j.cej.2021.133536>
4. C. Liu, P. Hu, Z. Xu, X. Ma, Z. Rao Experimental investigation on thermal properties of sodium acetate trihydrate based phase change materials for thermal energy storage. Thermochim. Acta **674**, 28–35 (2019). <https://doi.org/10.1016/j.tca.2019.02.002>
5. G. Fang, W. Zhang, M. Yu, K. Meng, X. Tan Experimental investigation of high performance composite phase change materials based on sodium acetate trihydrate for solar thermal energy storage. Sol. Energy Mater. Sol. Cells **234**, 111418 (2022). <https://doi.org/10.1016/j.solmat.2021.111418>
6. W. Sun, G. Liang, F. Feng, H. He, Z. Gao Study on sodium acetate trihydrate-expand graphite-carbon nanotubes composite phase change materials with enhanced thermal conductivity for waste heat recovery. J. Energy Storage **55**, 105857 (2022). <https://doi.org/10.1016/j.est.2022.105857>
7. W. Wu, G. Ye, G. Zhang, X. Yang Composite phase change material with room-temperature-flexibility for battery thermal management. Chem. Eng. J. **428**, 131116 (2022). <https://doi.org/10.1016/j.cej.2021.131116>
8. Q. Huang, X. Li, G. Zhang, Y. Wang, J. Deng et al., Pouch lithium battery with a passive thermal management system using form-stable and flexible composite phase change materials. ACS Appl. Energy Mater. **4**, 1978–1992 (2021). <https://doi.org/10.1021/acsaem.0c03116>
9. H. Lei, X. Wang, Y. Li, H. Xie, W. Yu Organic-inorganic hybrid phase change materials with high energy storage density based on porous shaped paraffin/hydrated salt/expanded graphite composites. Energy **304**, 132169 (2024). <https://doi.org/10.1016/j.energy.2024.132169>

[S10] X. Man, H. Lu, Q. Xu, C. Wang, Z. Ling, Preparation and thermal property enhancement of sodium acetate trihydrate-lithium chloride-potassium chloride expanded graphite composite phase change materials. Sol. Energy Mater. Sol. Cells **266**, 112695 (2024). <https://doi.org/10.1016/j.solmat.2024.112695>
